# Evidence supporting a cultural evolutionary theory of prosocial religions in contemporary workplace safety data

**DOI:** 10.1038/s41598-022-09322-6

**Published:** 2022-03-28

**Authors:** Yuqi Gu, Connie X. Mao, Tim Johnson

**Affiliations:** 1 Atkinson Graduate School of Management, Willamette University, Salem, OR 97301 USA; 2 Fox School of Business, Temple University, Philadelphia, PA 19122 USA; 3 Center for Governance and Public Policy Research, Willamette Univ., Salem, OR 97301 USA

**Keywords:** Cultural evolution, Social evolution

## Abstract

A prominent line of cultural evolutionary theory hypothesizes that religiously inspired prosocial behavior enhances the fecundity of pious groups, causing them to outcompete non-religious communities and spread their prosocial values. We present evidence concerning contemporary workplace safety, in the United States, that unexpectedly tested implications of this cultural evolutionary hypothesis. Avoiding workplace injury requires cooperation and injury influences fitness, thus cultural evolutionary theory would anticipate that religious communities should exhibit fewer workplace injuries. Indeed, we find that the proportion of a community adhering to a religion correlates negatively with rates of workplace injury in its private-sector establishments. This correlation emerges primarily when secular workplace safety authorities are not prominent, thus echoing evidence that religiously inspired prosocial behavior mainly occurs absent “earthly” sanctioning authorities. Furthermore, the percent of religiously affiliated individuals in a community correlates with safety investments, suggesting that workplace injury reductions in religious communities result from individually costly, group-benefitting cooperation.

## Introduction

The descriptive study of religion’s influence on social behavior dates back centuries^1,2^, yet only recently have researchers transitioned from gathering anecdotal^1,2^ and observational^3^ evidence to collecting experimental data^4^ and performing meta-analysis^5^ on the subject. This transition has yielded a reliable evidence base indicating that priming religious beliefs enhances prosocial behavior^5^ and self-control^6^—particularly among certain religious sections^7^. With this empirical foundation, theories^8^ have expanded on early efforts^9,10^ to surmise the large-scale social and evolutionary consequences of religion’s effect on behavior. The resulting picture proposes that prosocial religions catalyze large-scale cooperation in devout groups, thereby improving those groups’ fecundity and causing them to outcompete non-religious communities—which, in turn, spreads their values and triggers further iterations of this dynamic^8^. Research^8^ describes patterns of reproduction^11^, conversion^12^, and group persistence^10^ consistent with such an evolutionary model, and here we add to that body of evidence by showing another way that religion influences outcomes associated with greater fecundity. Specifically, we provide evidence that the degree of religious adherence across communities—that is, the percent of a community’s population who adhere to a religion—correlates negatively with those communities’ rates of workplace injury—a health outcome with fitness consequences in both small-scale^13^ and industrialized^14^ contexts. In addition to yielding evidence relevant to a prominent strain of cultural evolutionary theory, our investigation also reinforces recent demonstrations of cultural evolutionary theory’s applicability to contemporary public policy^15,16^ and it sheds new light on a means by which private governance might substitute for public regulation^17^.

Like the theory it tests, our study rests on evidence concerning religion’s effect on social behavior. Initial observational studies presented mixed evidence about the effect of religion on social behavior. On the one hand, past research found that religious devotion correlated with cooperation in common pool dilemmas^3^, that a community’s percent of adherents to a world religion related to giving in ultimatum games^18^, and that belief in a moralistic omniscient deity was associated with greater altruism toward co-religionists^19^. However, other observational studies found that religious orientations correlated with egoistic motivation in helping scenarios^20^, that self-reported religiosity ran orthogonal to giving in dictator games^4^, and that religious adherence did not correlate with either trust or public goods contributions^21^, though it might play a role in sustaining the latter behavior once it has occurred^22^. These mixed findings were clarified once researchers introduced the exogenous priming of religious concepts. When priming religious concepts, researchers found that religious ideation increased giving in dictator games^4^ and, generally, experimental studies found that exogenous exposure to religious constructs consistently enhanced prosocial behavior among the devout^5^. Later studies showed that religion’s effect on prosocial behavior can vary across denominations: focusing on affiliates of Christion religions, research found that religious priming increased cooperation among Protestants, whereas it decreased cooperation among Catholics in an experimental public goods game conducted in a laboratory setting^7^. Although this pattern of results might stem from the particular socio-cultural laboratory setting, it indicates that religious denominations may yield varying effects on social behavior. Religious primes also led individuals to endure discomfort, delay gratification, persist in tasks following exertion, and concentrate in the presence of conflicting information—that is, they caused individuals to exert self control^6^.

Evidence that priming religion spurs cooperation and self-control among certain religious sects has led to theories proposing that religious beliefs play a central role in human cooperation and order^23^—and such consequences might, in turn, lead to the promulgation of those very same religious beliefs^9,19^. For instance, altruistic and reciprocal caregiving among early Christians may have resulted in differential mortality during ancient plagues and, thus, greater reproductive rates among Christians than secular Romans^9^. More generally, a recently proposed strain of cultural evolution theory surmises that religion’s effect on prosocial behavior initiates a positive feedback cycle: religion induces cooperation among the religious, thus improving the religious community’s well-being and increasing its fertility, which leads its members to outcompete secular groups and spread the very values that improved cooperation initially^8^.

Observational data supports the contours of this theory. Ethnographic evidence suggests that Islamic practices resulted in material improvements that increased rates of conversion to Islam in Africa^12^. Demographic evidence indicates higher rates of fertility among the religiously affiliated^11^. Religious communes persist longer than secular ones^10^. Each of these pieces of evidence coincide with the expectations of a cultural evolutionary model in which religious groups’ pro-sociality increases their fecundity.

Here we add to that body of evidence by reporting findings that indicate the relationship between religious adherence and physical well-being—specifically, physical well-being in contemporary workplaces. Not only is workplace injury a modern socio-economic problem that imposes costs estimated to be greater than those of all cancers combined^24^, but it can influence reproduction through various pathways^14^. Such observations echo anthropological evidence indicating that debilitating injuries are common in small-scale societies and carry fitness consequences^13^. Our study thus sheds light on a new mechanism—improved safety—through which religious adherence affects a physical outcome known to influence fecundity^13,14^.

Albeit initially conceived without cultural evolutionary theory in mind, our study builds on a large body of recent research in finance^25–34^ and hypothesizes that religious adherence improves workplace safety. For one, religious adherence enhances the cooperation that workplace safety requires. Whereas personal-injury avoidance entails weighing the private benefits of avoidance against its costs^35^, workplace-safety activities require individuals to incur personal costs to keep others safe—that is, to create a benefit for others. Such incentives raise the possibility that safety measures are underprovided due to free-riding^36^. Religious adherence militates against such free-riding^8^, however, thus leading us to expect lower rates of worker injury in the presence of more religiously affiliated individuals. Also, religious adherence influences the self-control and risk aversion known to improve workplace safety^37^. Intuitively, impulsivity and a willingness to take risks on the job likely influence rates of workplace injury. An impulsive individual might spurn safety regulations on a whim; risk tolerant individuals might accept greater degrees of danger due to their personal constitution. In keeping with these possibilities, evidence suggests that conditions facilitating self-control lead to improvements in safety^38^ and workers’ revealed risk preferences correlate with the likelihood of injury^39^. When coupled with past research showing that religion bolsters self-control^6^ and religious individuals exhibit greater risk aversion^30,33^, those findings provide reason to expect that religious adherence will be associated with lower rates of workplace injury.

Using a diverse range of data sources, we tested those hypotheses in statistical models of the Total Case Rate (coded as TCR) calculated as “the number of recordable cases (defined as the sum of total number of deaths and all injuries and illnesses that results in days away from work or with job transfer or restriction, and other recordable cases) × 200,000/Employee hours worked”^40^, as well as the natural logarithm of the Total Cases (Ln(1 + TC)), defined as “total number of recordable cases (including deaths and all injuries and illnesses)” that OSHA recorded^40^. We found evidence of a correlation between communities’ degrees of religious adherence and their subsequent rates of workplace injury in private-sector establishments. This correlation appears to be driven by the degree of Protestant adherence in a community: a community’s degree of Protestant adherence correlates negatively with workplace injury, but no such relationship appears between Catholic adherence and workplace injury, thus coinciding with past evidence of greater cooperation among Protestants vis-à-vis Catholics^7^ in laboratory experiments. Furthermore, we report evidence suggesting that this relationship holds because of religion’s effect on cooperation and self-control. The percent of religiously affiliated individuals in a community correlates positively with safety investment, suggesting that workplace injury reductions in religious communities result from individually costly, group-benefitting cooperative actions. Also, as in behavioral experiments^41^, we find that these effects of religious adherence disappear in the presence of an “earthly” sanctioning authority—namely, strong labor unions—thus suggesting that religious norms act as a substitute for other forms of workplace safety regulation. Furthermore, communities that exhibit behaviors implying a greater tolerance for risk and weaker self-control show a more pronounced negative relationship between workplace injury and religious adherence, therefore suggesting that religious adherence substitutes for self-control in communities in which that personal attribute is lacking. Together, these findings provide a source of contemporary support in the United States for key predictions derived from a cultural evolutionary theory of prosocial religions^8^.

The findings, moreover, coincide with the results of new research^42^, which we learned of during the review of this paper, that report results similar to the findings we present below. Using comparable or the same data sets as those examined in our investigation, research found that a community’s religiosity influences both workplace safety and workplace safety measures in the private establishments within its borders^42^—essentially the same result we report below. Our findings overlap with those results, but our study places those findings in the context of cultural evolutionary theory and it also presents a further set of novel robustness tests. Specifically, our study assesses the correlation between workplace safety and religious adherence in the locality of a firm’s headquarters, as well as in the locality of its establishments. Furthermore, we assessed the hypothesis that religiosity correlates with self-control and risk aversion, and found evidence consistent with that hypothesis, thus suggesting an additional mechanism that might explain the correlation between religiosity and workplace safety outcomes. Also, in contrast with the previously published research on this subject, we found varying correlations between workplace safety and religious adherence across denominations, whereas previous research found no such difference^42^. Thus, in addition to presenting results that inadvertently provide a quasi-replication of recently published research, we also offer robustness checks and extensions that further illuminate the relationship between religious adherence and workplace safety.

## Results

### Community religious adherence and workplace injury

As presented in Table 1, regressing the total case rate (TCR) (viz. “the number of recordable cases (defined as the sum of total number of deaths and all injuries and illnesses that results in days away from work or with job transfer or restriction, and other recordable cases) × 200,000/Employee hours worked”)^40^, on the county’s proportion of residents acknowledging a religious adherence^30,33,34^ in the prior year results in a negative, precisely estimated coefficient on that focal predictor variable ($\hat{\beta }$ = − 2.73, *SE* = 0.69, *t* = − 3.98, 95% *CI* = [− 4.07, − 1.39]). Inclusion of covariates suggested by the past literature^34,43,44^ along with industry-by-year fixed effects (see “Methods” and Supplementary materials), changes the absolute magnitude of this coefficient, but it does not alter the estimated coefficient’s substantive or overall statistical interpretation ($\hat{\beta }$ = − 1.47, *SE* = 0.53, *t* = − 2.78, 95% *CI* = [− 2.41, − 0.43]). Substantively, when using the estimates from model (b) of Table 1 and moving from the first quartile (0.42) to the third quartile (0.57) of our focal predictor, TCR decreases by 0.22 ((0.57 − 0.42) *1.471), which is equivalent to 3.7% (0.22/5.96) of TCR’s median value. This magnitude is larger than that of other independent variables in our model, including key financial predictors such as assets (moving from the 1st to the 3rd quartile of assets increases TCR by 0.09), market to book ratio (− 0.04), FCF to assets ratio (0.10), cash to assets ratio (0.10) and dividend to assets ratio (− 0.03), and comparable to that of leverage (0.27). However, it remains smaller than that of sales to assets ratio (0.34), PPE to assets ratio (0.71) and capital expenditures (CAPEX) to assets ratio (0.32) (see Supplementary Information for coefficient values for all predictors).

**Table 1:** The relationship between the degree of county religious affiliation and measures of workplace injury.

Model index	(a)	(b)	(c)	(d)
Dependent variable	*TCR*_*t*+1_	*TCR*_*t*+1_	*Ln*(1 + *TC*)_*t*+1_	*Ln*(1 + *TC*)_*t*+1_
Proportion of residents adhere to a religion in counties where establishments are located	− 2.73*** [− 4.07, − 1.39] (0.69)	− 1.47*** [− 2.51, − 0.43] (0.53)	− 0.36*** [− 0.590, − 0.12] (0.12)	− 0.19** [− 0.31, − 0.06] (0.06)
Covariates and industry-by-year fixed effects	No	Yes	No	Yes
N	31,835	31,835	31,835	31,835
Adjusted R^2^	0.002	0.299	0.001	0.649

Columns (a) and (b) display statistics from models that regressed each private-sector establishment’s Total Case Rate of injuries, illnesses, and deaths (TCR)—as recorded by the United States Occupational Safety and Health Administration (OSHA)^40^ from 2002 to 2011—on, respectively, its county’s proportion of residents acknowledging a religious adherence^30,33,34^ (a) and that variable plus a set of covariates and industry-by-year fixed effects (b). Columns (c) and (d) report regressions of the natural logarithm of one plus Total Cases of injuries, illnesses, and deaths (TC)^40^ on those same variables. All coefficient estimates and associated statistics appear in the supplementary information, but we display the estimate for the focal coefficient—proportion of population adhere to a religion in the county where an establishment is located—in the third row of the table, following the model index and the listing of the dependent variable used in each model. The fourth row indicates whether the model involved covariates and binary indicators signaling the industry-year of a given record for an establishment. The second to last row lists the number of observations (N) and the final row displays the proportion of explained variance (R^2^), adjusted for covariates. Asterisks denote the level of *p* values: ***p < 0.01, **p < 0.05, *p < 0.10.

The relationship also holds when we log transform the total number of cases (*Ln*(1 + *TC*)) as our dependent variable (Table 1c–d) and regress that variable on the prior year’s proportion of religious individuals in the community. In an unconditional model, we can reject the null hypothesis that our focal predictor equals zero ($\hat{\beta } = - \,0.36, \;SE = 0.12$, $t = - \,3.01, \;95\% \;CI = \left[ { - \,0.59,\; - \,0.12} \right]$). The inclusion of covariates in the model shrinks the focal independent variable’s estimated coefficient along with its standard error ($\hat{\beta } = - \,0.19$, $SE = 0.06$, $t = - 2.97$, $95\% \;CI = \left[ { - \,0.31,\; - \,0.06} \right]$), thus leading us to again reject the null hypothesis that the coefficient equals zero. In the supplementary information, we report evidence consistent with these findings that results from an instrumental variable design that further rules out confounding and other sources of endogeneity.

### Religious adherence near headquarters versus establishments

Prior studies suggest that the degree of religious adherence in the county where a firm locates its headquarters can affect various corporate behaviors^25–28^, thus we examined whether the degree of religious adherence in the locality of corporate headquarters proves more important in explaining workplace injury rates than the degree of religious adherence in the communities where enterprise establishments (e.g., plants, see “Methods” section) are located. Table 2 displays model estimates suggesting that the degree of religious adherence surrounding an enterprise’s headquarters correlates with workplace injury in a different manner than the degree of religious adherence in the community surrounding an enterprise’s establishments. In the models reported in Table 2, we replaced our focal predictor from the models reported in Table 1a–d with the proportion of residents who adhere to a religion in the county where an enterprise is headquartered. We cannot reject the null hypothesis that the coefficient associated with the latter variable equals zero, except in our unconditional model that regresses TCR on the proportion of religious affiliates in the county of headquarters (Table 2a). However, the relationship between that variable and TCR is positive ($\hat{\beta } = 2.67$, $SE = 0.98$, $t = 2.73$, $95\% \;CI = \left[ {0.75,\;4.59} \right]$), thus conflicting with our expectation that religiously induced cooperation would reduce workplace injury. Accordingly, we included both the proportion of religious adherents surrounding establishments and the proportion of religious adherents surrounding headquarters as predictors in a subsequent set of models, thus allowing for relative comparison of their coefficient estimates (Table 3a–b). When regressing TCR on those predictors (Table 3a), we could reject the null hypothesis of a zero value for the coefficient associated with the degree of religious adherence in the counties in which an enterprise’s establishments are located ($\hat{\beta } = - \,1.49$, $SE = 0.54$, $t = - \,2.75$, $95\% \;CI = \left[ { - \,2.54,\; - \,0.43} \right]$), but we could not make the same decision for the coefficient associated with the degree of religious adherence in the counties in which an enterprise’s headquarter is located ($\hat{\beta } = 0.90$, $SE = 0.83$, $t = 1.08$, $95\% \; CI = \left[ { - \,0.73, \;2.52} \right]$). A comparable pattern of results appeared when regressing the natural logarithm of total cases on those predictors (Table 3b). These findings support the idea that religious adherence near the “ground floor” of enterprises, not the executive suite, correlates with workplace injury.

**Table 2:** The relationship between the degree of religious affiliation among residents in the county in which an enterprise’s *headquarters* is located and measures of workplace injury in its establishments.

Model index	(a)	(b)	(c)	(d)
Dependent variable	*TCR*_*t*+1_	*TCR*_*t*+1_	*Ln*(1 + *TC*)_*t*+1_	*Ln*(1 + *TC*)_*t*+1_
Proportion of residents adhere to a religion in counties where headquarters are located	2.67*** [0.75, 4.59] (0.98)	0.51 [− 1.09, 2.10] (0.82)	0.13 [− 0.23, 0.49] (0.19)	0.15 [− 0.05, 0.35] (0.10)
Covariates and industry-by-year fixed effects	No	Yes	No	Yes
N	31,662	31,623	31,662	31,623
Adjusted R^2^	0.001	0.299	< 0.001	0.649

Columns (a) and (b) display statistics from models that regressed each private-sector establishment’s Total Case Rate of injuries, illnesses, and deaths (TCR)—as recorded by the United States Occupational Safety and Health Administration (OSHA)^40^ from 2002 to 2011—on, respectively, its county’s proportion of residents acknowledging a religious adherence^30,33,34^ (a) and that variable plus a set of covariates and industry-by-year fixed effects (b). Columns (c) and (d) report regressions of the natural logarithm of one plus Total Cases of injuries, illnesses, and deaths (TC)^40^ on those same variables. All coefficient estimates and associated statistics appear in the supplementary information, but we display the estimate for the focal coefficient—proportion of population adhere to a religion in the county where an enterprise’s headquarter is located—in the third row of the table, following the model index and the listing of the dependent variable used in each model. The fourth row indicates whether the model involved covariates and binary indicators signaling the industry-year of a given record for an establishment. The second to last row lists the number of observations (N) and the final row displays the proportion of explained variance (R^2^), adjusted for covariates. Asterisks denote the level of *p* values: ***p < 0.01, **p < 0.05, *p < 0.10.

**Table 3:** The degree of religious affiliation in counties where establishments are located influences workplace injury rates more clearly than religious affiliation in the counties where an enterprise’s headquarters are located.

model index	(a)	(b)
Dependent Variable	*TCR*_*t*+1_	*Ln*(1 + *TC*)_*t*+1_
Proportion of residents adhere to a religion in counties where establishments are located	− 1.49*** [− 2.54, − 0.43] (0.54)	− 0.20*** [− 0.32, − 0.07] (0.06)
Proportion of residents adhere to a religion in counties where headquarters are located	0.90 [− 0.73, 2.52] (0.83)	0.20* [− 0.003, 0.41] (0.10)
Covariates and industry-by-year fixed effects	Yes	Yes
N	31,623	31,623
Adjusted R^2^	0.31	0.65

Column (a) displays statistics from a model that regressed each private-sector establishment’s Total Case Rate of injuries, illnesses, and deaths (TCR)—as recorded by the United States Occupational Safety and Health Administration (OSHA)^40^ from 2002 to 2011—on the proportion of residents acknowledging a religious adherence in its county and that in the county where its enterprise’s headquarter^30,33,34^ is located plus a set of covariates and industry-by-year fixed effects. Column (b) reports the statistics from a model that regressed each private-sector establishment’s natural logarithm of one plus Total Cases of injuries, illnesses, and deaths (TC)^40^ on those same variables. All coefficient estimates and associated statistics appear in the supplementary information, but we display the estimate for the focal coefficients—proportion of population adhere to a religion in the county where the establishment is located and that in the county where its enterprise’s headquarter is located—in the third row and fourth row of the table, following the model index and the listing of the dependent variable used in each model. The fifth row indicates whether the model involved covariates and binary indicators signaling the industry-year of a given record for an establishment. The second to last row lists the number of observations (N) and the final row displays the proportion of explained variance (R^2^), adjusted for covariates. Asterisks denote the level of *p* values: ***p < 0.01, **p < 0.05, *p < 0.10.

### Religious denomination and workplace injury

Prior literature shows that Protestants display greater risk-aversion^32,33^ and cooperation^7^ than Catholics. As a result, we computed the respective number of Protestant and Catholic religious adherents in counties local to the private-sector establishments we studied and we divided those counts by the counties’ total populations^33^. We, then, replicate the regressions in Table 1, albeit with the rates of Protestant and Catholic adherence, respectively, added to replace the focal predictor in the model. (In the supplementary information, we report regressions in which we serially add these variables, thus displaying the relationship between Protestant adherence and workplace injury sans controlling for Catholic adherence, the relationship between Catholic adherence and workplace injury absent control for Protestant adherence, then the relationship with both Protestant and Catholic religiosity added).

As shown in Table 4a–b, we find that the degree of Protestant adherence correlates negatively with TCR and Ln(1 + TC), whereas Catholic adherence appears orthogonal to those workplace injury outcomes. On the other hand, the coefficient estimate for the Catholic adherence variable nearly equals its associated standard error, thus resulting in a 95%-confidence interval that spans zero.

**Table 4:** Diverse relationships between the degree of affiliation with particular religious denominations and workplace injury.

Model index	(a)	(b)
Dependent variable	*TCR*_*t*+1_	*Ln*(1 + *TC*)_*t*+1_
Proportion of residents adhere to protestant religion	− 1.26*** [− 2.11, − 0.40] (0.44)	− 0.15*** [− 0.25, − 0.05] (0.05)
Proportion of residents adhere to catholic religion	0.60 [− 0.48, 1.67] (0.55)	0.10 [− 0.03, 0.22] (0.07)
Covariates and industry-by-year fixed effects	Yes	Yes
N	31,835	31,835
Adjusted R^2^	0.299	0.649

Column (a) of Table 4 displays statistics from models that regressed each private-sector establishment’s Total Case Rate of injuries, illnesses, and deaths (TCR)—as recorded by the United States Occupational Safety and Health Administration (OSHA)^40^ from 2002 to 2011—on its county’s proportion of residents acknowledging a religious adherence to, respectively, the Protestant Christian religion and the Catholic Christian religion^30,33,34^. Column (b) reports a regression of the natural logarithm of one plus Total Cases of injuries, illnesses, and deaths (TC)^40^ on those same variables. All models include covariates and industry-by-year fixed effects, with the complete coefficient estimates for all covariates reported in the supplementary information. The second to last row lists the number of observations (N) and the final row displays the proportion of explained variance (R^2^), adjusted for covariates. Asterisks denote the level of *p* values: ***p < 0.01, **p < 0.05, *p < 0.10.

### Religion’s effect on injuries in the presence of strong labor unions

Our findings suggest that the rate of religious adherence influences worker safety, just as past research indicates that religious adherence increases prosocial behaviors. However, the latter line of inquiry also reports that the effects of religious adherence become muted in the presence of “earthly” authorities that can sanction misbehavior^41^. In the context of our study, labor unions perform this “earthly” role by monitoring working conditions and threatening sanctions—such as work stoppages or lawsuits—if workplace conditions are unsafe. Accordingly, we divided our full sample into two groups, one with strong union power and the other with weak union power, then regressed in each subsample injury rates on local religious adherence and our list of covariates (all coefficient estimates appear in the supplementary information).

Two variables proxy for union power in these analyses and are used to divide the subsample. The first is *Union Membership*—the percent of employees in an industry, during a given year, who are members of a union e.g., see^45^. The second proxy is *Bargaining Agreement*—the percent of employees in an industry-year who are included in a collective bargaining agreement e.g., see^45^.We use each as a separate measure of union power. We partition our sample based on the sample medians of these two measures in a year.

When using Union Membership as a proxy for union strength and estimating our model under conditions of strong union power, we cannot reject the null hypothesis that local religious adherence does not correlate with TCR ($\hat{\beta } = - \,0.30$, $SE = 0.83$, $t = - \,0.36$, $p = 0.717$, $95\% \;CI = \left[ { - \,1.92, \;1.32} \right]$). However, in the sub-sample of establishments with low Union Membership, we observe a negative relationship between local religious adherence and TCR that reaches a magnitude unlikely to be observed in the data were the coefficient actually equal to zero ($\hat{\beta } = - \,2.77$, $SE = 0.62$, $t = - \,4.5$, $p < 0.001$, $95\% \;CI = \left[ { - \,3.98,\; - \,1.56} \right]$). A test of equality of the coefficients allows us to reject the hypothesis that they are the same (Wald Test, *χ*^2^ = 13.35, *p* < 0.001). A similar pattern of results occurs when using Bargaining Agreement as a proxy for union strength. We estimate a small coefficient with a 95% confidence interval spanning zero in the data subsample indicating strong union power according to the Bargaining Agreement measure ($\hat{\beta }$ = − 0.20, *SE* = 0.83, *t* = − 0.25, *p* = 0.81, 95% *CI* = [− 1.83, 1.42]), whereas in the low union power sample we estimate a coefficient indicating a negative relationship between local religious adherence and workplace injury that allows us to reject the null hypothesis of no correlation ($\hat{\beta }$ = − 2.88, *SE* = 0.62, *t* = − 4.67, *p* < 0.001, 95% *CI* = [− 4.09, − 1.67]). We can reject the null hypothesis of no difference in the coefficients across subsamples distinguished by the level of Bargaining Agreement (Wald Test, χ^2^ = 15.71, *p* < 0.001). These findings parallel evidence that non-supernatural sources of monitoring and sanctioning crowd out religion’s effect on prosocial behavior^41^: in the presence of strong unions that can scrutinize workplace safety and pressure non-compliant employers, local religious adherence yields little impact on workplace injury, whereas it substitutes for such activities in the presence of weak unions.

### Cooperation as a mechanism underlying religion’s relationship to workplace safety

Interpreting our findings in relation to a cultural evolutionary theory of prosocial religions rests on the premise that cooperation and self-control serve as the mechanisms through which local religious affiliates achieve lower rates of workplace injury. Evidence pertaining to the relationship between religion and cooperation in workplace safety can be gleaned from analysis of managerial investment. Investments in workplace safety by management constitute costly measures that yield benefit to workers and the enterprise overall, not to the individual manager who could alternatively use the spent resources to advance personal objectives ranging from plumped-up expense accounts to pandering demonstrations of financial austerity.

We use abnormal discretionary expenses per employee as a measure of workplace safety investment—as in past studies^44^—and, to ensure that our results are not a by-product of that measure, we test the robustness of our findings with a separate, firm-safety index resulting from an assessment of enterprises workplace health and safety programs (i.e. costly measures or equipment used to ensure workplace safety) (see “Methods” section). Because these measures depict firm-level activity (i.e. they are at the level of the firm, not the level of establishments in a county), we replaced our focal predictor with a firm-level variable that took the weighted average of the proportion of religious affiliates in each county in which a firm had establishments, in a given year, and weight by the number of employees in each establishment (see “Methods” section).

As shown in Table 5, when regressing abnormal discretionary expenses on this measure, we find a large coefficient, albeit paired with a substantial standard error ($\hat{\beta } = 20.93$, $SE = 10.73$, $t = 1.95$, $p = 0.051$), which prevents us from rejecting the null hypothesis at the 95% confidence level of a two-tailed test, albeit consistent with the hypothesis that religion increases investment (a one-tailed test would result in rejection of the null with 95% confidence). Adding a measure of the proportion of religious adherents in the county of headquarters further weakens the precision of the estimate for our measure of firm-level religious adherents ($\hat{\beta } = 19.83$, $SE = 10.89$, $t = 1.82$, $p = 0.069$), thus preventing rejection of the null hypothesis at the 95%-confidence level assuming a two-tailed test, but facilitating rejection of a one-tailed test—unlike the coefficient estimate for the share of religious adherents in the county of headquarters ($\hat{\beta } = 68.95$, $SE = 42.70$, $t = 1.61$, $p = 0.107$). As with all of our models, the supplementary materials provide a complete listing of the coefficient estimates and relevant statistics.

**Table 5:** The relationship between the degree of local religiosity on overall firm level workplace safety investment.

Model index	(a)	(b)	(c)	(d)	(e)	(f)
Dependent variable	*AbDisExp*_*t*_	*AbDisExp*_*t*_	*AbDisExp*_*t*_	*KLD Safety Index*_*t*_	*KLD Safety Index*_*t*_	*KLD Safety Index*_*t*_
Proportion of residents adhere to a religion in counties where establishments are located	20.931* [− 0.12, 41.98] (10.73)		19.828* [− 1.52, 41.18] (10.89)	4.406*** [1.45, 7.36] (1.51)		4.438*** [1.53, 7.35] (1.48)
Proportion of residents adhere to a religion at headquarters		74.900* [− 8.92, 158.72] (42.73)	68.949 [− 14.81, 152.71] (42.71)		− 2.711 [− 25.76, 20.33] (11.76)	− 3.703 [− 26.26, 18.85] (11.51)
Covariates and firm and year fixed effects	Yes	Yes	Yes	Yes	Yes	Yes
N	5966	5890	5890	3369	3344	3344
Adjusted R^2^ or Pseudo R^2^	0.808	0.808	0.808	0.574	0.568	0.570

Columns (a)–(c) displays statistics from OLS models that regressed each enterprise’s abnormal discretionary expenses per employee^44^ on, respectively, its weighted average value of the religiosities of the counties where the enterprise’s establishments are located, weighted by the number of employees in each establishment (a) and the proportion of residents acknowledging a religious affiliation in county where its enterprise’s headquarter is located (b) and both religiosity measures (c)^30,33,34^. A set of covariates and firm and year fixed effects are included in all models. Columns (d)–(f) report ordered logit regressions of the safety index—constructed using the “MSCI ESG KLD STATS” data set from MSCI ESG Research—on those same variables. All coefficient estimates and associated statistics appear in the supplementary information, but we display the estimate for the focal coefficients—weighted average establishment religiosity and headquarter religiosity—in the third and fourth row of the table, respectively, following the model index and the listing of the dependent variable used in each model. The fifth row indicates whether the model involved covariates and binary indicators signaling the firm and year of a given record for an enterprise. The second to last row lists the number of observations (N) and the final row displays the proportion of explained variance (R^2^), adjusted for covariates. Asterisks denote the level of *p* values: ***p < 0.01, **p < 0.05, *p < 0.10.

Regressing the safety index on the firm-level measure of local religious adherence and controls from our previous models indicates a positive relationship between that outcome variable and focal predictor ($\hat{\beta } = 4.41$, $SE = 1.51$, $t = 2.92$, $p = 0.004$). Adding the degree of religious adherence at firm headquarters as a predictor to the aforementioned model does little to change the size and statistical interpretation of our key predictor of local religious adherence at the firm-level ($\hat{\beta } = 4.44$, $SE = 1.48$, $t = 2.99$, $p = 0.003$).

### Self-control and risk aversion as mechanisms underlying religion’s relationship to workplace safety

Past research also suggests that priming religion increases self-control^6^ and religious individuals exhibit risk aversion^30,33^. Self-control facilitates the maintenance of social order generally^8^ and should yield positive workplace safety outcomes. Likewise, risk aversion should reduce an individual’s willingness to engage in dangerous activities on the job, thus improving workplace safety outcomes.

Both factors, however, might vary across communities independent of religiosity, thus religious adherence would act as a substitute mechanism for self-control when that attribute is lacking in the population. To consider this possibility, we gathered evidence on another realm in which self-control and risk avoidance is important—namely, automobile safety—and we used rates of auto accidents (state-level “fatality rate per 100 million vehicle miles traveled”) from the National Highway Traffic Safety Administration (NHTSA)^46^ as a proxy for overall self-control and risk aversion in the population. We divided our sample into two groups—those above and below the median auto accident rate in the state where an establishment is located—and replicated the models from Table 1 on each subsample. As shown in Table 6, when studying the sub-sample of communities with above-median accident rates (in which we would expect religiously induced self-control to substitute for control lacking in the community), we find that regressing TCR on our focal predictor results in estimates that allow us to reject the null hypothesis of no relationship between outcome and predictor ($\hat{\beta } = - \,1.70$, $SE = 0.73$, $t = - \,2.33$, $p = 0.02$). Estimates from the same regression performed on communities with below-median accident rates does not result in rejection of the null hypothesis ($\hat{\beta } = - \,0.98$, $SE = 0.72$, $t = - \,1.38$, $p = 0.17$). When modeling the logarithm level of workplace injuries, we find comparable results. In the sub-sample of communities with above-median accident rates, a regression of ln(1 + TC) on our focal predictor results in estimates that allow us to reject the null hypothesis of no relationship between outcome and predictor ($\hat{\beta } = - \,0.25$, $SE = 0.09$, $t = - 2.94$, $p = 0.003$), but estimates generated from data concerning communities with below-median accident rates does not result in rejection of the null hypothesis ($\hat{\beta } = - \,0.06, \;SE = 0.09,\; t = - \,0.70,\; p = 0.48$). These results are consistent with the hypothesis that religiosity increases self-control and risk aversion, thus the degree of local religious adherence correlates negatively with workplace injury because religiosity boosts self-control and risk aversion when secular drivers of those traits are absent.

**Table 6:** The relationship between religiosity and workplace safety conditional on auto accident rate in the state where the establishment is located.

Model index	(a)	(b)	(c)	(d)
Subsample	High auto accident rate	Low high auto accident rate	High auto accident rate	Low high auto accident rate
Dependent variable	*TCR*_*t*+1_	*TCR*_*t*+1_	*Ln*(1 + *TC*)_*t*+1_	*Ln*(1 + *TC*)_*t*+1_
Proportion of residents adhere to a religion in counties where establishments are located	− 1.700** [− 3.13, − 0.27] (0.73)	− 0.989 [− 2.40, 0.42] (0.72)	− 0.253*** [− 0.42, 0.08] (0.09)	− 0.061 [− 0.22, 0.11] (0.09)
Covariates and industry-by-year fixed effects	Yes	Yes	Yes	Yes
N	16,094	15,741	16,094	15,741
Adjusted R^2^	0.273	0.330	0.647	0.655

Columns (a) and (b) display statistics from models that regressed each private-sector establishment’s Total Case Rate of injuries, illnesses, and deaths (TCR)—as recorded by the United States Occupational Safety and Health Administration (OSHA) ^40^ from 2002 to 2011—on its county’s proportion of residents acknowledging a religious affiliation^30,33,34^ in the subsample where the state-level *Auto Accident Rate* is above or equal to (a) or below (b) the sample median in a year, where *Auto Accident Rate* is the “fatality rate per 100 million vehicle miles traveled”, as defined by Unites States Department of Transportation^46^. Columns (c) and (d) report regressions of the natural logarithm of one plus Total Cases of injuries, illnesses, and deaths (TC)^40^ on those same variables. All coefficient estimates and associated statistics appear in the supplementary information, but we display the estimate for the focal coefficient—proportion of county population adhere to a religion—in the fourth row of the table, respectively, following the model index, subsample indicator and the listing of the dependent variable used in each model. The fifth row indicates whether the model involved covariates and binary indicators signaling the industry-by-year of a given record for an establishment. The second to last row lists the number of observations (N) and the final row displays the proportion of explained variance (R^2^), adjusted for covariates. Asterisks denote the level of *p* values: ***p < 0.01, **p < 0.05, *p < 0.10.

## Discussion

Our findings provide contemporary evidence relevant to hypotheses derived from a cultural evolutionary theory of prosocial religions^8^. Focusing on the policy-relevant context of workplace safety, we find that the proportion of religious individuals in a community correlates negatively with its rate of worker injury—as would be expected if religious constructs compel cooperative activities that yield improvements to the physical well-being of group members. Also, consistent with cultural evolutionary theory, we find evidence that this relationship results from cooperative costly investments in group-beneficial safety measures and religiously induced self-control and risk aversion that substitutes for those traits when they are lacking in the wider population. Moreover, we observe a correlation between the degree of religious adherence in a community and workplace safety only in environments with weak union power—a result that echoes past research showing that religiously inspired prosocial behavior mainly occurs absent “earthly” sources of monitoring and sanctioning^41^. Although these findings dovetail with a number of dimensions of a cultural evolutionary theory of prosocial religion and they are consistent with the research that we discovered during the review of this manuscript^42^, they also warrant caution.

First, the findings result from observational data analyses, thus they must be regarded as correlational. Although we attempted to establish causal connections and rule out confounding factors, our efforts do not offer clean causal inferences that might result from a study that could experimentally manipulate the focal variables of interest. For instance, it is possible that correlations between collectivism and religious participation might account for the association between religious adherence and workplace safety that we observe (we thank an anonymous reviewer for pointing out this particular confounding factor). Unfortunately, we lack an adequate measure of collectivism across the time periods and communities in our study, thus future research will need to explore this possibility. As a result, we recommend a conservative interpretation of our findings as correlations.

Second, our study considers extra-individual phenomena and, thus, it does not test how individual-level processes generate the outcomes being observed. Like the theory we test, we focus on how the degree of group religious adherence influences group prosociality and, in turn, group outcomes. For example, a correlation between religious adherence and personality traits associated with conscientiousness might underlie our finding (we thank an anonymous reviewer for this great point). Again, our study cannot examine that possibility. Future research will need to collect and analyze data at the individual level to understand how individual psychological processes and activities within groups yield the group cooperation that lowers workplace injury rates.

Third, our study draws on a diverse range of data sources; though this might allow for testing a broader range of hypotheses and performing additional robustness checks, it also means that potential incongruity in the alignment of data with our unit of analysis is possible. Though we took measure to avoid such problems, diverse data collection efforts lend themselves to the possibility of subtle differences in how the collectors of each data set defined the unit of analysis.

Fourth, religious attendance and adherence may have declined in the U.S. over the time span of our study, thus raising the possibility that our findings result spuriously from coincident declines in religiosity and workplace injury. Including year fixed effects in our regression models can mitigate some of the concern surrounding this possible source of spurious correlation; however, a stronger test to address the concern will be identifying exogenous drivers of religious adherence and testing their relation to workplace injury rates, which presents a fruitful future research avenue for assessing whether a genuine link between religious adherence and workplace safety exists.

Finally, the evidence we present results from a study that did not set out to test the hypotheses of cultural evolutionary theory; rather, it was an applied investigation^47^ whose hypotheses about religion, cooperation, and workplace safety were derived independent of cultural evolutionary theory and only ex-post were the implications for cultural evolutionary theory recognized. On the one hand, this feature of the study has the disadvantage of any research whose design was not deliberately crafted to test hypotheses derived from a given theory; on the other hand, the study did not run the risk of crafting its design in a way favorable to the theory whose hypotheses it happened to test. In any event, those interpreting this research should know that it initially did not set out to test the predictions of cultural evolutionary theory.

With those caveats noted, not only do the results of this investigation offer evidence relevant to cultural evolutionary theories, but they shed light on a major public policy issue—workplace injury—which results in economic consequences in excess of the costs of all cancers^24^. Indeed, the current paper contributes to recent research that studies how private-sector enterprises implement voluntary restrictions as substitutes for public regulation^48–50^. Past research has noted that private regulatory initiatives generally serve a strategic purpose and are promulgated from the top down because they enhance reputation^51–53^, prevent tougher public regulations^17,54,55^, yield compromises among ideologically disparate interests^56^, placate activists^54,57^, ensure self-determination^58^, advance neo-liberalism^59^, and improve competitiveness^60–62^. However, in this paper we have shown that individuals might also carry out de facto private regulations in a decentralized, non-strategic manner and these activities can be predicted based on theoretical postulates in basic research concerning cultural evolution.

In this way, our research continues past efforts^63^ to explore the contemporary implications of cultural evolutionary processes. Here we have reported evidence consistent with one such implication: cultural evolutionary processes may foster religions that induce prosocial behaviors that influence contemporary outcomes related to fitness, such as workplace safety.

## Methods

### Data

We analyzed data from “Occupational Safety Health Administration” (OSHA)^64^ annual surveys taken among 80,000 private-sector establishments every year from 1996 through 2011. These surveys were part of OSHA’s Data Initiative Program (ODI)^64^. As described in past work^42,65^ and OSHA’s rules^64^, OSHA required establishments with 11 + employees at a facility to document all “work-related injuries and illnesses”, and OSHA officials were provided access to this documentation. An establishment is defined as “a single physical location where business is conducted or where services or industrial operations are performed. For activities where employees do not work at a single physical location, such as construction; transportation; communications, electric, gas and sanitary services; and similar operations, the establishment is represented by main or branch offices, terminals, stations, etc. that either supervise such activities or are the base from which personnel carry out these activities”^66^. Industries with “low occupational injury and illness rates” received exemption from these documentation efforts^42,64,65^. The data was then used by OSHA to calculate establishment-specific “case rates”^42,64,65^. OSHA data also contains establishment information such as “Establishment name, address, industry, average number of employees, average number of working hours, (whether or not the facility experienced), strikes or lockout, shutdown or layoff, or natural disasters”^67^. Following prior research, we restricted our sample period to 2002–2011 due to a change in OSHA’s recordkeeping rule and associated forms in 2002, making it hard to compare the injury and illness variables before and after the change^65^.

Next, we matched each establishment in the OSHA data set to observations in the Compustat data set created by Wharton Research Data Service manually using the name of the company. After excluding companies in financial service sector and utility sector, as in the prior literature^43,44^, our prime sample consisted of 78,305 observations for establishment-specific injury rates during a year. These observations are from 23,562 unique establishments and 4357 unique firms. On average, firms in the sample have 3.4 establishments.

Consistent with existing research^42^, county-level religious adherence data were obtained from the “Church and Church Membership files” collected by the “Association of Religion Data Archive” (ARDA)^68^. We merged OSHA’s data set to the ARDA data set, with enterprises’ financial data retrieved from the Compustat database created by Wharton Research Data Service. We fused these merged files with U.S. Census Bureau’s county-level demographic data and state-level auto accident and fatality rate data from the “National Highway Traffic Safety Administration” (NHTSA)^46^, plus industry-level union membership data from Unionstats.com^69^. Our final sample size is comprised of a maximum of 31,835 establishment-year observations from 11,002 establishments and 1923 companies. Variation in the sample size by analysis results from missing values. Consistent with other studies of the effect of religiosity on private enterprise activity, we Winsorize all non-count variables, by replacing the top 1% of the data with the value at the 99th percentile and bottom 1% of the data with the value at the 1st percentile to reduce the disproportionate effect of extreme outliers. Our main results are qualitatively and statistically similar if we do not Winsorize these variables, save for one analysis concerning safety investment (see Supplementary Information).

### Variable construction

As in previous work using the same OSHA data^65^, whose description we echo in this paragraph, we adopt OSHA’s definition of incidence rates of occupational injuries and illness and employ two measures of rate of worker injuries. The first measurement, total case rate (coded as TCR), is calculated as “the number of recordable cases (defined as the sum of total number of deaths and all injuries and illnesses that results in days away from work or with job transfer or restriction, and other recordable cases) × 200,000/Employee hours worked”^40^. The second measure, Total Case (TC), is the “total number of recordable cases (including deaths and all injuries and illnesses)” as defined above^40^.

We follow existing literature and measure a community’s share of religious adherents by its county’s proportion of residents acknowledging a religious adherence^30,33,34^. Since this information is available for every 10 years, we follow previous literature^30,33,34^ and linearly interpolate religiosity measures between years 2000 and 2010.

In our regressions, we follow past research and control for a complete vector of covariates at firm-, establishment- and county-level^43,44^. Echoing the description of previous research using these same data, we include—at the firm level—ln(Assets), the logarithm of assets; LEV, computed as the ratio book value of debt to total assets; PPETA, net property, plant, and equipment scaled by total assets; SALETA, the ratio of revenue to total assets; CAPEXTA, measured by the ratio of capital expenditure to total assets; MTB, the ratio of market value of assets to the book value of assets; FCFTA, total free cash flows scaled by total assets; CASHTA, cash or equivalent scaled by total assets; DIVTA, common dividend paid by the firm during the year scaled by total assets. At establishment level, we include Ln(EMP), the logarithm of “average number of employees (working in a given establishment during the year)"^67^; HRS, measured by the “total number of working hours”^67^ divided by EMP^67^ in a given establishment; STRIKE, a binary variable for “(whether there is) a strike or lockout”, or otherwise^67^; SHUT, a binary variable for “(whether there is) a shutdown or layoff”, or otherwise^67^; SEASON, which signals whether the establishment “employs seasonal workers” via a binary indicator^67^; DISASTER, which takes a value of 1 if “adverse weather conditions or natural disasters” affected an establishment^67^. We also include in the model demographic characteristics of the county as in past research^34^. Specifically, as in the prior study, we used the following variables: Log(POP)^34^, the logarithm of “the total population in the county”^34^; Log(INCOMEAVG), the logarithm of the “per capita personal income of the county”^34^; EDU, “the proportion of county population above age 25 that has completed a bachelor’s degree or higher”^34^; M/F, “the number of males divided by the number of females” in the county^34^; and Minority, the proportion of all individuals in the county identifying with a race other than non-Hispanic white^34^. Since county-level demographic data are from census which takes place every 10 years, we linearly interpolate the demographic measures variables for years with missing information^34^. Further detail concerning our variable definitions appears in the supplementary information.

### Statistical methods: community religious adherence and workplace injury

We first assess the effect of local religiosity on workplace safety in a multivariate framework by estimating the following equation using the OLS regressions:

1 $$\begin{aligned} TCR_{i, t + 1} \,or\, Ln\left( {1 + TC} \right)_{i, t + 1} & = \alpha + \beta\, Religiosity_{EMi, t}\; or\; Religiosity_{HQi, t} \\ & \quad + \gamma^{\prime}\; Controls_{i, t} + Industry_{i} *Year_{t} + \varepsilon_{i,t,} \\ \end{aligned}$$

where *i* indicates establishment and *t* indicates year. *Religiosity*_*EM*_ or *Religiosity*_*HQ*_ is the proportion of residents acknowledging a religious adherence in a county where the establishment or headquarter is located^30,33,34^, respectively. Following past research^43,44^, we include a complete vector of firm- and establishment-level covariates that may relate to worker safety as described above and displayed in SI Table 1. At the county level, we also control for a vector of demographic characteristics where the establishment is located using variables included in past research^34^. To control for unobservable, time-varying industry-level characteristics, we further include fixed effects for each industry-year group (i.e. dummy variables that take a value of one when an establishment in a year resides in a given industry, and zero otherwise). In our main tests, all independent variables are measured at year t-1.

### Statistical methods: religious adherence near headquarters versus establishments

Prior literature focuses on firm-level analysis by examining how the degree of religious adherence in a county where a firm’s headquarter is located can affect various corporate behaviors^25–28^. The granularity of our establishment-level data allows us to precisely match the injury rate data to the county where religious adherence data is available, which offers a significant advantage of our paper. Such a dataset allows us to investigate how and to what extent worker safety policy is shaped by headquarters (top management) vs. factors at the establishment (e.g., plant-level managers or workers’ norms)) and to shed light on how safety decisions are delegated within the corporate hierarchy. Accordingly, we computed the fraction of residents acknowledging a religious adherence in a county where the headquarter is located^30,33,34^ and we regressed our measures of workplace safety on that variable, along with the covariates described in the previous section.

### Statistical methods: religious denomination and workplace injury

We computed the respective number of Protestant and Catholic religious adherents in counties local to the private-sector establishments we studied and we divided those counts by the counties’ total populations^30,33,34^. We, then, replicated the regressions from the section, Community Religious Adherence and Workplace Injury, Table1 with the focal predictor removed and the rates of Protestant and Catholic adherence, respectively, added to the model in the focal predictor’s place.

### Statistical methods: religion’s effect on injuries in the presence of strong labor unions

We partition our full sample into two subgroups, one with strong union power and the other with weak union power; then, in each subsample, we regressed our measures of workplace injury on local religious adherence and our set of covariates and fixed effects. Two variables proxy for union power in these analyses and are used to divide the subsample. The first is *Union Membership*—the percent of employees in an industry, during a given year, who are members of a union e.g., see^45^. The second proxy is Bargaining Agreement—the percent of employees in an industry-year who are included in a collective bargaining agreement e.g., see^45^. Previous research^45^ combines these measures, though we treat them as separate variables that represent union power. We partition our sample based on the sample medians of these two measures in a year.

### Statistical methods: cooperation as a mechanism underlying religion’s relationship to workplace safety

We focus on firm-level analysis since data on safety investments are unavailable at the establishment-level. Nevertheless, firm-level safety investments may reflect aggregated spending on safety resulting from individual plant-level managerial decisions that are shaped by local religious beliefs around each establishment. It is challenging to measure the firms’ investments in workplace safety because it involves not only direct expenses, such as replacing aging equipment with safer models, but also expenditures on intangible activities “such as safety training and program, safety incentives, and safety consulting”^70^. We construct two variables to proxy for firm level workplace safety investment. Following past work^44^, we proxy investments in safety using abnormal discretionary expenses per employee, which is estimated based on previous work^71^. Additionally, for robustness, we also construct an alternatively firm-level safety performance measure based on the ratings from the “MSCI ESG KLD STATS” data set from MSCI ESG Research, which is the largest provider of environmental, social and governance research^72^. This data set presents a binary (i.e., 0/1) summary of “strength” and “concern” on ESG ratings, including health and safety. We take the rating of “Health and Safety Strength” and subtract that of “Health and Safety Concern” to construct the KLD Safety Index, following prior research^65^. A higher index value therefore indicates better safety performance. Assuming a firm’s overall safety performance is a result of corporate investments in tangible and intangible assets strengthening safety, we expect the safety index to serve as a reasonable proxy for safety investment.

To conduct firm-level analysis, we aggregate establishment-level religiosity into a firm-level variable, which is defined as the weighted-average value of the proportion of religious affiliates in the county of all establishments of a firm in a year, weighted by establishment employee headcount. We regress the aforementioned measures of safety investment on both this variable and the set of covariates following past research^73,74^.

### Statistical methods: self-control and risk aversion as mechanisms underlying religion’s relationship to workplace safety

We gathered rates of auto accidents (state-level “fatality rate per 100 million vehicle miles traveled”) from the NHTSA^46^ and divided our sample into two groups—those above and those below the median auto accident rate in the state where an establishment is located. Then we replicated our baseline model described above in the section, Community Religious Adherence and Workplace Injury, in each subsample.

## Supplementary Information

Supplementary Information.

## Data Availability

Data used in this study included sensitive commercial information released by OSHA only under agreement to C.X.M. and proprietary data derived from commercial data sets such as Compustat. Researchers can obtain the data via application to the relevant parties; those data can then be used with our computer code to organize the data in the manner prepared for our analysis.

## Code availability

We have supplied all of the computer code that we used to organize and analyze the data. Analyses were conducted in Stata/MP Version 14.
