# Supplementary material for: Evidence supporting a cultural evolutionary theory of prosocial religions in contemporary workplace safety data
Source: Sci Rep. 2022 Mar 28;12:5239. doi: 10.1038/s41598-022-09322-6 (PMC8960878; doi:10.1038/s41598-022-09322-6)
Supplement: Supplementary file 1 — Supplementary Information. [file 41598_2022_9322_MOESM1_ESM.docx]

**Supplementary Information**

**Evidence supporting a cultural evolutionary theory of prosocial religions in contemporary workplace safety data**

**Yuqi Gu, Connie X. Mao, and Tim Johnson**

**Contents**

[1. Introduction to the Supplementary Information 3](#_Toc96110484)

[2. Further Details on Data and Sample Selection 4](#_Toc96110485)

[3. Further Details on Variable Construction 5](#_Toc96110486)

[SI Table 1 – Variable Definitions. 6](#_Toc96110487)

[SI Table 2 – Summary Statistics 8](#_Toc96110488)

[4. Replication of Main Results without Winsorizing 9](#_Toc96110489)

[SI Table 3 – Summary Statistics without Winsorizing Variables 10](#_Toc96110490)

[SI Table 4 – Community Religious Adherence and Workplace Injury without Winsorizing Variables 11](#_Toc96110491)

[SI Table 5 – Community Religious Adherence Near Establishments versus Headquarters and Workplace Injury without Winsorizing Variables 12](#_Toc96110492)

[SI Table 6 – Religious Denomination and Workplace Injury without Winsorizing Variables 13](#_Toc96110493)

[SI Table 7 – Religion’s Effect on Injuries in the Presence of Strong Labor Unions without Winsorizing Variables 14](#_Toc96110494)

[SI Table 8–Cooperation as a Mechanism Underlying Religion’s Relationship to Workplace Safety without Winsorizing Variables 15](#_Toc96110495)

[SI Table 9 – Self-Control and Risk Aversion as Mechanisms Underlying Religion’s Relationship to Workplace Safety without Winsorizing Variables 16](#_Toc96110496)

[5. Complete Model Coefficient Estimates 17](#_Toc96110497)

[5.1. Community Religious Adherence and Workplace Injury 18](#_Toc96110498)

[SI Table 10 – Community Religious Adherence and Workplace Injury 20](#_Toc96110499)

[5.2. Religious Adherence near Headquarters Versus Establishments 24](#_Toc96110500)

[SI Table 11. Religious Adherence near Headquarters versus Establishments 25](#_Toc96110501)

[5.3. Religious Denomination and Workplace Injury 27](#_Toc96110502)

[SI Table 12. Religious Denomination and Workplace Injury 28](#_Toc96110503)

[5.4. Religion’s Effect on Injuries in the Presence of Strong Labor Unions 30](#_Toc96110504)

[SI Table 13. Religion’s Effect on Injuries in the Presence of Strong Labor Unions 31](#_Toc96110505)

[5.5. Cooperation as a Mechanism underlying Religion’s Relationship to Workplace Safety. 33](#_Toc96110506)

[SI Table 14. Cooperation as A Mechanism Underlying Religion’s Relationship to Workplace Safety 34](#_Toc96110507)

[5.6. Self-Control and Risk Aversion as Mechanisms underlying Religion’s Relationship to Workplace Safety 36](#_Toc96110508)

[SI Table 15. Self-Control and Risk Aversion as Mechanisms Underlying Religion’s Relationship to Workplace Safety 37](#_Toc96110509)

[6. Addressing Endogeneity via Instrumental Variable Analyses 39](#_Toc96110510)

[SI Table 16 – Instrumental Variable Analysis of Community Religiosity and Workplace Safety 41](#_Toc96110511)

SI Table 17 – Instrumental Variable Analysis of Religious Adherence near Headquarters and Workplace Safety……………………………………………………………………………..45

[Supplementary Information References 48](#_Toc96110512)

# 1. Introduction to the Supplementary Information

In this supplementary information file, we provide greater detail concerning (i) the data and sample selection of our investigation, (ii) the methods used in our analyses, (iii) the results obtained from those methods, and (iv) the robustness of those findings. To facilitate readers’ interpretation of this content, we fully contextualize all information in narrative form. Although this approach leads to redundancy (i.e. repetition of information from the main text in the supplementary information), it ensures that the supplementary information can be interpreted without frequent reference to the main text. One difference between the main text and the supplementary information file concerns our use of variable monikers instead of longhand descriptions. We use those monikers because a key feature of this supplementary information file is the presentation of all coefficient estimates in our regression models; providing those estimates means that we must have a concise means to depict those various covariates and using shorthand labels for them facilitates does so. All variable labels are presented in italics and, as discussed below, we present a table that pairs each variable moniker with a detailed description of the variable it describes.

The supplementary information follows the standard structure of an article in the social sciences. It first presents information about the data and construction of variables in the study. Then, it presents the most basic form of analyses, with more sophisticated approaches presented subsequently to gauge the robustness of data. We hope this format provides readers a convenient way to learn more from our study.

# 2. Further Details on Data and Sample Selection

We analyzed data from “Occupational Safety Health Administration” (OSHA)^1^ annual surveys taken among 80,000 private-sector establishments every year from 1996 through 2011. These surveys were part of OSHA’s Data Initiative Program (ODI). As described in past work^2,3^ and OSHA’s rules^1^, OSHA required establishments^[[1]](#footnote-1)^ with 11+ employees at a facility to document all “work-related injuries and illnesses”, and OSHA officials were provided access to this documentation. An establishment is defined as “a single physical location where business is conducted or where services or industrial operations are performed. For activities where employees do not work at a single physical location, such as construction; transportation; communications, electric, gas and sanitary services; and similar operations, the establishment is represented by main or branch offices, terminals, stations, etc. that either supervise such activities or are the base from which personnel carry out these activities”^4^. Industries with “low occupational injury and illness rates” received exemption from these documentation efforts^1-3^. The data was then used by OSHA to calculate establishment-specific “case rates” ^1-3^. OSHA data also contains establishment information such as “Establishment name, address, industry, average number of employees, average number of working hours, (whether or not the facility experienced), strikes or lockout, shutdown or layoff, or natural disasters”^5^. Following prior research, we restricted our sample period to 2002-2011 due to a change in OSHA’s recordkeeping rule and associated forms in 2002, making it hard to compare the injury and illness variables before and after the change^3^. The data were obtained by one of the authors (C.X.M.) from the OSHA via an agreement.

Next, we matched each establishment in the OSHA data set to observations in the Compustat data set created by Wharton Research Data Service manually using the name of the company. After excluding companies in financial service sector and utility sector, following prior literature^6,7^, our prime sample is consists of 78,305 observations for establishment-specific injury rates during a year. These observations are from 23,562 unique establishments and 4,357 unique firms. On average, firm in the sample have 3.4 establishments.

Consistent with existing research^2^, county-level religious adherence data is obtained from the “Church and Church Membership files” collected by the “Association of Religion Data Archive” (ARDA)^8^. We merged OSHA’s data set to ARDA data set, with enterprise’s financial data retrieved from the “Compustat database” created by “Wharton Research Data Service”. We fused these merged files with U.S. Census Bureau’s county-level demographic data and state-level auto accident and fatality rate data from the “National Highway Traffic Safety Administration” (NHTSA)^9^, plus industry-level union membership data from Unionstats.com^10^. Our final sample size is comprised of a maximum of 31,835 establishment-year observations from 11,002 establishments and 1,923 companies.

The data did not receive IRB review because all data used in the study concerned organizations, not individual people, and no observations concerning individual humans took place. Although data used here cannot be shared by the authors due to their proprietary nature, the authors will help any prospective users in the process of identifying and requesting the proper data for replication purposes. Also, all code used in the study will be made available to users so that it can be inspected and used in the replication of the study’s results.

# 3. Further Details on Variable Construction

As in previous work using the same OSHA data^3^, whose description we echo in this paragraph, we adopt OSHA’s definition of incidence rates of occupational injuries and illness and employ two measures of rate of worker injuries. The first measurement, total case rate (coded as TCR), is calculated as “the number of recordable cases (defined as the sum of total number of deaths and all injuries and illnesses that results in days away from work or with job transfer or restriction, and other recordable cases) × 200,000 / Employee hours worked” ^11^. The second measure, Total Case (TC), is the “total number of recordable cases (including deaths and all injuries and illnesses)” as defined above^11^.

We follow existing literature and measure a community’s share of religious affiliates by its county’s proportion of residents acknowledging a religious adherence^12-14^. Since this information is available for every 10 years, we follow previous literature^12-14^ and linearly interpolate religiosity measures between years 2000 and 2010.

In our regressions, we follow past research and control for a complete vector of covariates at firm-, establishment- and county-level^6,7^. Echoing the description of previous research using these same data, we include—at the firm level—Ln(Assets), the logarithm of assets; LEV, computed as the ratio book value of debt to total assets; PPETA, net property, plant, and equipment scaled by total assets; SALETA, the ratio of revenue to total assets; CAPEXTA, measured by the ratio of capital expenditure to total assets; MTB, the ratio of market value of assets to the book value of assets; FCFTA, total free cash flows scaled by total assets; CASHTA, cash or equivalent scaled by total assets; DIVTA, common dividend paid by the firm during the year scaled by total assets. At establishment level, we include Ln(EMP), the logarithm of “average number of employees (working in a given establishment during the year)"^5^; HRS, measured by the “total number of working hours” ^5^ divided by EMP ^5^ in a given establishment; STRIKE, a binary variable for “(whether there is) a strike or lockout”, or otherwise^5^; SHUT, a binary variable for “(whether there is) a shutdown or layoff”, or otherwise^5^; SEASON, which signals whether the establishment “employs seasonal workers” via a binary indicator^5^; DISASTER, which takes a value of 1 if “adverse weather conditions or natural disasters” affected an establishment^5^. We also include in the model demographic characteristics of the county used in past research^14^. Specifically, as in the prior study, we used the following variables: Log(POP)^14^, the logarithm of “the total population in the county”^14^; Log(INCOMEAVG), the logarithm of the “per capita personal income of the county”^14^; EDU, “the proportion of county population above age 25 that has completed a bachelor’s degree or higher”^14^; M/F, “the number of males divided by the number of females” in the county^14^; and Minority, the proportion of all individuals in the county identifying with a race other than non-Hispanic white^14^. Since county-level demographic data are from census which takes place every 10 years, we linearly interpolate the demographic measures variables for years with missing information^14^.

For ease of reference, a complete description of study variables appears in SI Table 1, below, and we follow that table with a presentation of the summary statistics for variables used in SI Table 2. As mentioned in the main text, we report analyses in which we Winsorize all non-count variables, by replacing top 1% of the data with the value at the 99th percentile and bottom 1% of the data with the value at the 1st percentile, respectively to reduce the disproportionate effect of extreme outliers in our main text. As a result, we report in SI Tables 3 – 9 the results of our analyses without Winsorizing, as described below.

**SI Table 1 – Definitions of Variables**

|  | **Definition** | | |
| --- | --- | --- | --- |
| **Establishment Characteristics** | | | |
| *TCR* | “The number of recordable cases (defined as the sum of total number of deaths and all injuries and illnesses that results in days away from work or with job transfer or restriction, and other recordable cases) × 200,000 / Employee hours worked” ^11^. | | |
| *TC* | “Total number of recordable cases (including deaths and all injuries and illnesses)”^11^. | | |
| *EMP* | “Average number of employees (working in a given establishment during the year)"^5^. | | |
| *HRS* | Total “number of working hours”^5^ scaled by “average number of employees”^5^. | | |
| *STRIKE* | A binary variable for “(whether there is) a strike or lockout”, or otherwise^5^. | | |
| *SHUT* | A binary variable for “(whether there is) a shutdown or layoff”, or otherwise^5^ | | |
| *SEASON* | A binary indicator that signals whether the establishment “employs seasonal workers”^5^. | | |
| *DISASTER* | A binary indicator that signals whether “adverse weather conditions or natural disasters” affected an establishment^5^. | | |
| **Firm-level** |  | | |
| *Assets* | The book value of assets. | | |
| *LEV* | Book value of debt scaled by total assets | | |
| *PPETA* | Net property, plant, and equipment (PPE) scaled by total assets | | |
| *SALETA* | Total sales divided by end-of-year total assets. | | |
| *CAPEXTA* | Annual capital expenditure scaled by end-of-year total assets. | | |
| *MTB* | Total market value of assets to the end-of-year book value of assets. | | |
| *FCFTA* | Free cash flows scaled by end-of-year assets. Free cash flow is computed as (oibdq-xint-txdi-capx). | | |
| *CASHTA* | Cash or equivalent scaled by end-of-year total assets. | | |
| *DIVTA* | The ratio of common dividend paid by the firm during the year to end-of-year total assets. | | |
| *KLD Safety Index* | Firm-level safety performance measure based on the ratings from the “MSCI ESG KLD STATS” data set from MSCI ESG Research Is index is computed as the rating of “Health and Safety Strength” minus the rating of “Health and Safety Concern”^3^. | | |
| *AbDisExp* | The abnormal discretionary expenses per employee as defined in prior study^7^. In particular, it is “the residual from the  following model which is estimated for firm i in year t within each two-digit SIC code/year with at least 15 observations:  $\frac{{SGA}_{i,t}}{{EMP}_{i,t-1}}=\beta_{0}+\beta_{1}\frac{1}{{EMP}_{i,t-1}}+\beta_{2}\frac{{Sales}_{i,t-1}}{{EMP}_{i,t-1}}+\varepsilon_{it}$  where SGA is the total selling, general, and administrative expenditures.” ^7^ | | |
| *KZ* | An index measuring a firm’s financial constraints, introduced by prior study^15^. | | |
| *ROA* | Operating income before depreciation scaled by end-of-year total assets. | | |
| *Macro Q* | Market value of total assets scaled by the PPE at the beginning of the year. | | |
| *Cash flow* | The sum of income before extraordinary items and depreciation and amortization scaled by the PPE at the beginning of the year. | | |
| **Industry-Specific Variable** | | |  |
| *Union Membership* | The percent of workers belonging to unions in an industry during a year^16^ | | |
| *Bargain Agreement* | The percent of employees within an industry covered by collective agreement (i.e., collective bargaining agreement) during a year^16^ | | |
| **County-Specific Variable** | |  | |
| *Religiosity* | The county’s proportion of residents acknowledging a religious adherence^12-14^ | | |
| *Protestant* | The county’s proportion of residents acknowledging a religious adherence to the Protestant Christian religion^12-14^ | | |
| *Catholic* | The county’s proportion of residents acknowledging a religious adherence to the Catholic Christian religion^12-14^ | | |
| *POP* | County population^14^ | | |
| *INCOMEAVG* | The “per capita personal income of the county”^14^ | | |
| *EDU* | “The proportion of county population above age 25 that has completed a bachelor’s degree or higher”^14^ | | |
| *M/F* | The number of male residents in the county scaled by that of female residents^14^ | | |
| *Minority* | The county’s proportion of residents identifying with a race other than non-Hispanic white^14^ | | |
| *Deadly Disaster* | A binary indicator that signals whether there is a disaster-related death in the county, as defined by SHELDUS^17^ | | |
| **State-Specific Variable** |  | | |
| *Auto Accident Rate* | The state-level “fatality rate per 100 million vehicle miles traveled”, as defined by Unites States Department of Transportation^9^ | | |

### SI Table 2 – Summary Statistics

Summary statistics for establishment-year characteristics. The sample includes all establishments from Data Initiative program collected by OSHA. Sample period is 2002 - 2011. Companies in financial service sector and utility sector are excluded, following prior literature^6,7^. Detailed definitions of covariates are shown in SI Table 1.

| Variable | N | Average | Standard  Deviation | 25^th^  Percentile | Median | 75^th^  Percentile |
| --- | --- | --- | --- | --- | --- | --- |
| *TCR* | 31,835 | 7.79 | 7.44 | 2.06 | 5.96 | 11.36 |
| *TC* | 31,835 | 24.07 | 85.85 | 3.00 | 8.00 | 20.00 |
| *Religiosity_EM_* | 31,835 | 0.50 | 0.11 | 0.42 | 0.50 | 0.57 |
| *Religiosity_HQ_* | 31,623 | 0.52 | 0.08 | 0.49 | 0.53 | 0.57 |
| *Assets* (in $billions) | 31,835 | 23.46 | 45.83 | 0.92 | 6.79 | 26.39 |
| *LEV* | 31,835 | 0.27 | 0.18 | 0.14 | 0.25 | 0.36 |
| *PPETA* | 31,835 | 0.37 | 0.20 | 0.20 | 0.35 | 0.55 |
| *SALETA* | 31,835 | 1.41 | 0.73 | 0.89 | 1.24 | 1.76 |
| *CAPEXTA* | 31,835 | 0.06 | 0.04 | 0.03 | 0.04 | 0.08 |
| *MTB* | 31,835 | 1.75 | 0.86 | 1.10 | 1.50 | 2.27 |
| *FCFTA* | 31,835 | 0.06 | 0.08 | 0.02 | 0.06 | 0.10 |
| *CASHTA* | 31,835 | 0.09 | 0.11 | 0.02 | 0.05 | 0.11 |
| *DIVTA* | 31,835 | 0.01 | 0.02 | 0.00 | 0.01 | 0.02 |
| *EMP* (in 000s) | 31,835 | 0.30 | 0.48 | 0.08 | 0.14 | 0.29 |
| *HRS* (in 000s) | 31,835 | 1.90 | 0.32 | 1.68 | 1.95 | 2.09 |
| *STRIKE* | 31,835 | 0.00 | 0.04 | 0.00 | 0.00 | 0.00 |
| *SHUT* | 31,835 | 0.09 | 0.29 | 0.00 | 0.00 | 0.00 |
| *SEASON* | 31,835 | 0.04 | 0.19 | 0.00 | 0.00 | 0.00 |
| *DISASTER* | 31,835 | 0.01 | 0.08 | 0.00 | 0.00 | 0.00 |
| *POP* (in 000s) | 31,835 | 860.24 | 1550.75 | 94.55 | 338.89 | 896.72 |
| *INCOMEAVG*(in 000s) | 31,835 | 24.16 | 5.68 | 20.15 | 23.33 | 26.92 |
| *EDU (%)* | 31,835 | 25.70 | 9.73 | 17.80 | 25.63 | 31.62 |
| *M/F* | 31,835 | 0.96 | 0.04 | 0.94 | 0.96 | 0.98 |
| *Minority* | 31,835 | 0.30 | 0.20 | 0.13 | 0.27 | 0.45 |

# 4. Replication of Main Results without Winsorizing

To ensure that extreme values of our study variables did not influence the results of our investigation, we report analyses in which we Winsorize all non-count variables, by replacing top 1% of the data with the value at the 99th percentile and bottom 1% of the data with the value at the 1st percentile, respectively to reduce the disproportionate effect of extreme outliers. To demonstrate our findings are robust absent Winsorizing, we report in SI Tables 3 – 9 the results of our analyses without Winsorizing. The qualitative and statistical interpretation of our findings remains the same for all analyses, except for analysis concerning safety investment in SI Table 8.

### SI Table 3 – Summary Statistics without Winsorizing Variables

| Variable | N | Average | Standard  Deviation | 25^th^  Percentile | Median | 75^th^  Percentile |
| --- | --- | --- | --- | --- | --- | --- |
|  |  |  |  |  |  |  |
| *TCR* | 31,835 | 7.81 | 7.56 | 2.06 | 5.96 | 11.36 |
| *TC* | 31,835 | 24.07 | 85.85 | 3.00 | 8.00 | 20.00 |
| *Religiosity_EM_* | 31,835 | 0.50 | 0.12 | 0.42 | 0.50 | 0.57 |
| *Religiosity_HQ_* | 31,623 | 0.52 | 0.08 | 0.49 | 0.53 | 0.57 |
| *Assets* (in $billions) | 31,835 | 23.46 | 45.83 | 0.92 | 6.79 | 26.39 |
| *LEV* | 31,835 | 0.28 | 0.21 | 0.14 | 0.25 | 0.36 |
| *PPETA* | 31,835 | 0.37 | 0.20 | 0.20 | 0.35 | 0.55 |
| *SALETA* | 31,835 | 1.41 | 0.75 | 0.89 | 1.24 | 1.76 |
| *CAPEXTA* | 31,835 | 0.06 | 0.04 | 0.03 | 0.04 | 0.08 |
| *MTB* | 31,835 | 1.77 | 1.76 | 1.10 | 1.50 | 2.27 |
| *FCFTA* | 31,835 | 0.05 | 0.31 | 0.02 | 0.06 | 0.10 |
| *CASHTA* | 31,835 | 0.09 | 0.12 | 0.02 | 0.05 | 0.11 |
| *DIVTA* | 31,835 | 0.01 | 0.03 | 0.00 | 0.01 | 0.02 |
| *EMP* (in 000s) | 31,835 | 5.06 | 1.09 | 4.34 | 4.98 | 5.69 |
| *HRS* (in 000s) | 31,835 | 1908.37 | 472.05 | 1683.46 | 1953.14 | 2086.65 |
| *STRIKE* | 31,835 | 0.00 | 0.04 | 0.00 | 0.00 | 0.00 |
| *SHUT* | 31,835 | 0.09 | 0.29 | 0.00 | 0.00 | 0.00 |
| *SEASON* | 31,835 | 0.04 | 0.19 | 0.00 | 0.00 | 0.00 |
| *DISASTER* | 31,835 | 0.01 | 0.08 | 0.00 | 0.00 | 0.00 |
| *POP* (in 000s) | 31,835 | 862.47 | 1563.45 | 94.55 | 338.89 | 896.72 |
| *INCOMEAVG*(in 000s) | 31,835 | 24.23 | 5.90 | 20.15 | 23.33 | 26.92 |
| *EDU (%)* | 31,835 | 25.75 | 9.89 | 17.80 | 25.63 | 31.62 |
| *M/F* | 31,835 | 0.96 | 0.04 | 0.94 | 0.96 | 0.98 |
| *Minority* | 31,835 | 0.30 | 0.20 | 0.13 | 0.27 | 0.45 |

### SI Table 4 – Community Religious Adherence and Workplace Injury without Winsorizing Variables

Panel A. Establishment-level religiosity

| Dependent Variable | *TCR_t+1_* | *TCR_t+1_* | *TCR_t+1_* | *TCR_t+1_* | *Ln(1+TC)_t+1_* | *Ln(1+TC)_t+1_* | *Ln(1+TC)_t+1_* | *Ln(1+TC)_t+1_* |
| --- | --- | --- | --- | --- | --- | --- | --- | --- |
|  | (1) | (2) | (3) | (4) | (5) | (6) | (7) | (8) |
| *Religiosity_EM_* | -2.643*** | -1.445** | -1.474*** | -1.489*** | -0.336*** | -0.173** | -0.180*** | -0.177*** |
|  | (0.658) | (0.594) | (0.526) | (0.522) | (0.115) | (0.071) | (0.061) | (0.061) |

Panel B. Headquarter-level religiosity

| Dependent Variable | *TCR_t+1_* | *TCR_t+1_* | *TCR_t+1_* | *TCR_t+1_* | *Ln(1+TC)_t+1_* | *Ln(1+TC)_t+1_* | *Ln(1+TC)_t+1_* | *Ln(1+TC)_t+1_* |
| --- | --- | --- | --- | --- | --- | --- | --- | --- |
|  | (1) | (2) | (3) | (4) | (5) | (6) | (7) | (8) |
| *Religiosity_HQ_* | 2.767*** | 3.068*** | 1.098 | 0.962 | 0.177 | 0.392*** | 0.174* | 0.160 |
|  | (0.963) | (0.958) | (0.845) | (0.839) | (0.186) | (0.118) | (0.105) | (0.104) |

### SI Table 5 – Community Religious Adherence Near Establishments versus Headquarters and Workplace Injury without Winsorizing Variables

| Dependent Variable | *TCR_t+1_* | *Ln(1+TC)_t+1_* |
| --- | --- | --- |
|  | (1) | (2) |
| *Religiosity_EM_* | -1.569*** | -0.186*** |
|  | (0.529) | (0.061) |
| *Religiosity_HQ_* | 1.381 | 0.210** |
|  | (0.851) | (0.104) |

### SI Table 6 – Religious Denomination and Workplace Injury without Winsorizing Variables

| Dependent  Variable | *TCR_t+1_* | *Ln(1+TC)_t+1_* | *TCR_t+1_* | *Ln(1+TC)_t+1_* | *TCR_t+1_* | *Ln(1+TC)_t+1_* |
| --- | --- | --- | --- | --- | --- | --- |
|  | (1) | (2) | (3) | (4) | (5) | (6) |
| *Protestant_EM_* | -1.620*** | -0.188*** |  |  | -1.382*** | -0.154*** |
|  | (0.411) | (0.047) |  |  | (0.446) | (0.052) |
| *Catholic_EM_* |  |  | 1.513*** | 0.189*** | 0.680 | 0.096 |
|  |  |  | (0.518) | (0.061) | (0.560) | (0.066) |

### SI Table 7 – Religion’s Effect on Injuries in the Presence of Strong Labor Unions without Winsorizing Variables

|  | Union Membership | | Bargain Agreement | |
| --- | --- | --- | --- | --- |
|  | High | Low | High | Low |
| Dependent Variable | *TCR_t+1_* | *TCR_t+1_* | *TCR_t+1_* | *TCR_t+1_* |
|  | (1) | (2) | (3) | (4) |
| *Religiosity_EM_* | -0.267 | -2.811*** | -0.163 | -2.926*** |
|  | (0.815) | (0.593) | (0.812) | (0.598) |

### SI Table 8–Cooperation as a Mechanism Underlying Religion’s Relationship to Workplace Safety without Winsorizing Variables

| Dependent Variable | *AbDisExp_t_* | *AbDisExp_t_* | *AbDisExp_t_* | *KLD Safety Index_t_* | *KLD Safety Index_t_* | *KLD Safety Index_t_* |
| --- | --- | --- | --- | --- | --- | --- |
|  | (1) | (2) | (3) | (4) | (5) | (6) |
| *Religiosity_EM_Aggregated_* | **16.288** |  | **15.482** | 4.208*** |  | 4.234*** |
|  | **(12.049)** |  | **(12.225)** | (1.407) |  | (1.388) |
| *Religiosity_HQ_* |  | 61.674 | 57.242 |  | -2.405 | -3.376 |
|  |  | (45.092) | (45.118) |  | (11.833) | (11.606) |

***Note.*** Coefficient estimates and standard errors in bold font result from analyses without Winsorizing that led to a different decision about rejection of the null hypothesis than analyses performed after Winsorizing variables.

### SI Table 9 – Self-Control and **Risk Aversion as Mechanisms Underlying Religion’s Relationship to Workplace Safety without Winsorizing Variables**

|  | Auto Accident Rate | | Auto Accident Rate | |
| --- | --- | --- | --- | --- |
|  | High | Low | High | Low |
| Dependent Variable | *TCR_t+1_* | *TCR_t+1_* | *Ln(1+TC)_t+1_* | *Ln(1+TC)_t+1_* |
|  |  |  |  |  |
|  | (1) | (2) | (3) | (4) |
| *Religiosity_EM_* | -1.595** | -1.118 | -0.240*** | -0.059 |
|  | (0.740) | (0.691) | (0.085) | (0.080) |

# 5. Complete Model Coefficient Estimates

In order to focus readers on the key statistical information relevant to an assessment of hypotheses derived from a cultural evolutionary theory of prosocial religions^18^, our manner of presenting findings in the main text presented statistical information solely related to the focal variable in our analysis. Here, in the supplementary information, we present the coefficient estimates and standard errors for other covariates included in our regression models. To properly contextualize our analyses, we introduce each table of findings with a narrative description of the results that echoes the results section of the main text. This redundancy hopefully reduces the frequency with which one needs to page back-and-forth form the main text to the supplementary information.

## 5.1. Community Religious Adherence and Workplace Injury

We first assess the effect of local religious adherence on enterprise’s worker safety in a multivariate framework by estimating the following equation using OLS regressions:

${TCR}_{i, t+1} or {Ln\left( 1+TC \right)}_{i, t+1}=\alpha+\beta{Religiosity_{EM}}_{i, t} or {Religiosity_{HQ}}_{i, t}+\gamma^{'}{Controls}_{i, t}+{Industry}_{i}*{Year}_{t}+\varepsilon_{i,t,}$ (1)

where *i* indicates establishment and *t* indicate year. *Religiosity_EM_* or *Religiosity_HQ_* is the proportion of residents acknowledging a religious adherence in a county where the establishment or headquarter is located^12-14^, respectively. Following past research^6,7^, we include a complete vector of firm- and establishment-level covariates that may relate to worker safety as described above and displayed in SI Table 1. At the county level, we also control for a vector of demographic characteristics where the establishment is located using variables included in past research^14^. To control for unobservable, time-varying industry-level characteristics, we further include fixed effects for each industry-year group (i.e. dummy variables that take a value of one when an establishment in a year resides in a given industry, and zero otherwise). In our main tests, all independent are measured at year t-1.We Winsorize all non-count variables by replacing top 1% of the data with the value at the 99^th^ percentile and bottom 1% of the data with the value at the 1^st^ percentile to reduce the disproportionate effect of extreme outliers, consistent with other studies of the effect of religiosity on private enterprise activity^19^.

We report the regression results in SI Table 10. In columns (1) and (5) of Panel A, we include the independent variable *Religiosity_EM_* as the only regressor. In columns (2) and (6), we add firm- and establishment-level variables as additional controls. In columns (3) and (7), we further include fixed effects for each industry-year group. In columns (4) and (8), county-level demographic characteristics are included as additional covariates. The coefficient estimates of *Religiosity_EM_* are significantly negative at the 5% level in all eight models. This evidence suggests that higher degree of establishment-level religious adherence is associated with a significantly lower injury incidence rate as well as lower number of total cases. The findings support the hypothesis that the degree of local religious adherence improves worker safety.

The coefficient estimates of firm-level characteristic variables in Panel A are mostly consistent with the findings in past research^6,7^. *TCR* is positively associated with *LEV*, *PPETA*, *SALETA*, and *CAPEXTA*; and *TCR* is negatively related to *DIVTA*. With respect to establishment-level controls, consistent with prior literature^3^, *TCR* is positively related to *Ln(EMP)* and *SEASON*, but negatively related to *HRS*. With respect to county-level controls, *TCR* is positively related to *Log(POP)* in a county and *M/F*, but negatively related to *EDU* and *Minority* presence.

In Panel A of SI Table 4, we estimate the same sets of OLS modelss but without Winsorizing variables. As with the results in Panel A of SI Table 10, *Religiosity_EM_* is negatively associated with injury rate and total case count without Winsorizing.

Prior literature focuses on firm-level analysis by examining how the degree of religious adherence in a county where a firm’s headquarter is located can affect various corporate behaviors^20-23^. The granularity of our establishment-level data allows us to precisely match the injury rate data to the county where religious adherence data is available, which offers a significant advantage of our paper. Such a dataset allows us to investigate how and to what extent worker safety policy is shaped by headquarters (top management) vs. factors at the establishment (e.g., plant-level managers or workers’ norms) and shed light on how safety decisions are delegated within the corporate hierarchy.

To this end, we examine in Panel B of SI Table 10 how *Religiosity_HQ_*, computed as the proportion of residents acknowledging a religious adherence in a county where the headquarter is located^12-14^, is related to workplace injury rate. We find that *Religiosity_HQ_* is insignificantly related to injury rate in six out of the eight models. The exceptions are in columns (1), (2) and (6), the coefficient estimates of *Religiosity_HQ_* are significantly positive.

### SI Table 10 – Community Religious Adherence and Workplace Injury

This table shows the results estimating the effect of religiosity local to establishment (Panel A) and to firm headquarter (Panel B) on workplace injury rates using establishment-level observations in OLS regressions. *TCR* is calculated as “the number of recordable cases (defined as the sum of total number of deaths and all injuries and illnesses that results in days away from work or with job transfer or restriction, and other recordable cases) × 200,000 / Employee hours worked” ^11^. *TC* is the “total number of recordable cases (including deaths and all injuries and illnesses)” as defined above^11^. *Religiosity_EM_* is the proportion of residents acknowledging a religious adherence in a county where the establishment is located^12-14^. *Religiosity_HQ_* is the religiosity measure in a county where the firm’s headquarter is located^12-14^. Detailed definitions of all other covariates are shown in SI Table 1. Standard errors are clustered by establishments and reported underneath coefficient estimates. Asterisks denote the level of p-values: *** *p*<0.01, ** *p*<0.05, **p*<0.10.

Panel A. Establishment-level religiosity

| Dependent Variable | *TCR_t+1_* | *TCR_t+1_* | *TCR_t+1_* | *TCR_t+1_* | *Ln(1+TC)_t+1_* | *Ln(1+TC)_t+1_* | *Ln(1+TC)_t+1_* | *Ln(1+TC)_t+1_* |
| --- | --- | --- | --- | --- | --- | --- | --- | --- |
|  | (1) | (2) | (3) | (4) | (5) | (6) | (7) | (8) |
| *Religiosity_EM_* | -2.730*** | -1.252** | -1.488*** | -1.471*** | -0.357*** | -0.176** | -0.192*** | -0.189*** |
|  | (0.685) | (0.591) | (0.525) | (0.530) | (0.119) | (0.073) | (0.063) | (0.064) |
| *Ln(Assets)* |  | 0.034 | 0.041 | 0.027 |  | -0.003 | -0.021*** | -0.025*** |
|  |  | (0.042) | (0.048) | (0.049) |  | (0.005) | (0.006) | (0.006) |
| *LEV* |  | 0.800** | 1.205*** | 1.258*** |  | 0.177*** | 0.199*** | 0.207*** |
|  |  | (0.405) | (0.400) | (0.399) |  | (0.052) | (0.050) | (0.050) |
| *PPETA* |  | 8.204*** | 2.180*** | 2.007*** |  | 1.143*** | 0.166** | 0.125* |
|  |  | (0.531) | (0.550) | (0.543) |  | (0.064) | (0.069) | (0.068) |
| *SALETA* |  | 0.817*** | 0.464*** | 0.390*** |  | 0.201*** | 0.098*** | 0.082*** |
|  |  | (0.096) | (0.124) | (0.125) |  | (0.012) | (0.015) | (0.015) |
| *CAPEX_Assets* |  | 4.100** | 6.450*** | 6.445*** |  | 0.928*** | 1.229*** | 1.217*** |
|  |  | (2.073) | (2.292) | (2.278) |  | (0.265) | (0.294) | (0.293) |
| *MTB* |  | -0.145* | -0.059 | -0.027 |  | -0.020* | 0.002 | 0.010 |
|  |  | (0.079) | (0.079) | (0.079) |  | (0.010) | (0.011) | (0.011) |
| *FCFTA* |  | 2.447*** | 1.254* | 1.173 |  | 0.255** | 0.126 | 0.109 |
|  |  | (0.702) | (0.721) | (0.715) |  | (0.101) | (0.102) | (0.101) |
| *CASHTA* |  | 2.923*** | 0.702 | 1.108 |  | 0.000 | -0.259*** | -0.177* |
|  |  | (0.672) | (0.679) | (0.681) |  | (0.092) | (0.093) | (0.092) |
| *DIVTA* |  | -17.393*** | -0.502 | -1.629 |  | -1.354** | 0.796 | 0.558 |
|  |  | (4.007) | (4.036) | (4.040) |  | (0.559) | (0.566) | (0.563) |
| *Ln(EMP)* |  | 0.156** | 0.214*** | 0.231*** |  | 0.851*** | 0.865*** | 0.869*** |
|  |  | (0.071) | (0.065) | (0.064) |  | (0.009) | (0.008) | (0.008) |
| *HRS* |  | -0.005*** | -0.004*** | -0.004*** |  | -0.000*** | -0.000 | -0.000 |
|  |  | (0.000) | (0.000) | (0.000) |  | (0.000) | (0.000) | (0.000) |
| *STRIKE* |  | 2.021* | 1.485 | 1.489 |  | 0.297* | 0.250* | 0.253* |
|  |  | (1.133) | (1.042) | (1.041) |  | (0.158) | (0.142) | (0.145) |
| *SHUT* |  | 0.780*** | 0.969*** | 0.960*** |  | 0.115*** | 0.167*** | 0.166*** |
|  |  | (0.200) | (0.183) | (0.183) |  | (0.023) | (0.021) | (0.021) |
| *SEASON* |  | 0.325 | 0.413* | 0.378 |  | 0.079*** | 0.050* | 0.045* |
|  |  | (0.254) | (0.232) | (0.232) |  | (0.029) | (0.026) | (0.026) |
| *DISASTER* |  | -0.277 | 0.212 | 0.287 |  | -0.079 | -0.022 | -0.018 |
|  |  | (0.581) | (0.531) | (0.535) |  | (0.062) | (0.056) | (0.056) |
| *Log(POP)* |  |  |  | 0.249*** |  |  |  | 0.027*** |
|  |  |  |  | (0.066) |  |  |  | (0.008) |
| *Log(INCOMEAVG)* |  |  |  | -0.393 |  |  |  | -0.131* |
|  |  |  |  | (0.607) |  |  |  | (0.072) |
| *EDU* |  |  |  | -0.046*** |  |  |  | -0.007*** |
|  |  |  |  | (0.013) |  |  |  | (0.002) |
| *M/F* |  |  |  | 3.493** |  |  |  | 0.444** |
|  |  |  |  | (1.679) |  |  |  | (0.199) |
| *Minority* |  |  |  | -2.528*** |  |  |  | -0.368*** |
|  |  |  |  | (0.431) |  |  |  | (0.052) |
| Constant | 9.165*** | 12.731*** | 13.032*** | 12.326** | 2.363*** | -2.377*** | -2.187*** | -1.340* |
|  | (0.355) | (0.777) | (0.752) | (5.855) | (0.061) | (0.092) | (0.092) | (0.697) |
| Industry-by-year    fixed effects | No | No | Yes | Yes | No | No | Yes | Yes |
| Observations | 31,835 | 31,835 | 31,835 | 31,835 | 31,835 | 31,835 | 31,835 | 31,835 |
| Adjusted R^2^ | 0.002 | 0.145 | 0.293 | 0.299 | 0.001 | 0.559 | 0.643 | 0.649 |

Panel B. Headquarter-level religiosity

| Dependent Variable | *TCR_t+1_* | *TCR_t+1_* | *TCR_t+1_* | *TCR_t+1_* | *Ln(1+TC)_t+1_* | *Ln(1+TC)_t+1_* | *Ln(1+TC)_t+1_* | *Ln(1+TC)_t+1_* |
| --- | --- | --- | --- | --- | --- | --- | --- | --- |
|  | (1) | (2) | (3) | (4) | (5) | (6) | (7) | (8) |
| *Religiosity_HQ_* | 2.668*** | 2.688*** | 0.578 | 0.505 | 0.133 | 0.408*** | 0.154 | 0.150 |
|  | (0.979) | (0.924) | (0.818) | (0.815) | (0.185) | (0.117) | (0.105) | (0.104) |
| *Ln(Assets)* |  | 0.040 | 0.053 | 0.040 |  | -0.002 | -0.020*** | -0.024*** |
|  |  | (0.043) | (0.049) | (0.049) |  | (0.005) | (0.006) | (0.006) |
| *LEV* |  | 0.945** | 1.335*** | 1.394*** |  | 0.198*** | 0.219*** | 0.227*** |
|  |  | (0.401) | (0.396) | (0.394) |  | (0.052) | (0.050) | (0.050) |
| *PPETA* |  | 8.185*** | 2.210*** | 2.031*** |  | 1.135*** | 0.161** | 0.120* |
|  |  | (0.531) | (0.553) | (0.546) |  | (0.063) | (0.069) | (0.068) |
| *SALETA* |  | 0.845*** | 0.491*** | 0.425*** |  | 0.206*** | 0.103*** | 0.087*** |
|  |  | (0.097) | (0.124) | (0.125) |  | (0.012) | (0.015) | (0.015) |
| *CAPEX_Assets* |  | 5.335** | 7.234*** | 7.247*** |  | 1.137*** | 1.395*** | 1.380*** |
|  |  | (2.078) | (2.329) | (2.317) |  | (0.267) | (0.299) | (0.298) |
| *MTB* |  | -0.175** | -0.062 | -0.031 |  | -0.025** | 0.001 | 0.009 |
|  |  | (0.078) | (0.079) | (0.078) |  | (0.010) | (0.011) | (0.011) |
| *FCFTA* |  | 2.651*** | 1.469** | 1.389* |  | 0.277*** | 0.149 | 0.134 |
|  |  | (0.700) | (0.717) | (0.710) |  | (0.101) | (0.102) | (0.100) |
| *CASHTA* |  | 3.432*** | 0.978 | 1.370** |  | 0.079 | -0.210** | -0.131 |
|  |  | (0.667) | (0.660) | (0.661) |  | (0.092) | (0.092) | (0.091) |
| *DIVTA* |  | -19.279*** | -2.030 | -3.165 |  | -1.575*** | 0.612 | 0.374 |
|  |  | (3.957) | (3.955) | (3.947) |  | (0.553) | (0.558) | (0.553) |
| *Ln(EMP)* |  | 0.148** | 0.202*** | 0.222*** |  | 0.850*** | 0.864*** | 0.868*** |
|  |  | (0.071) | (0.065) | (0.064) |  | (0.009) | (0.008) | (0.008) |
| *HRS* |  | -0.005*** | -0.004*** | -0.004*** |  | -0.000*** | -0.000 | -0.000 |
|  |  | (0.000) | (0.000) | (0.000) |  | (0.000) | (0.000) | (0.000) |
| *STRIKE* |  | 1.997* | 1.448 | 1.448 |  | 0.294* | 0.246* | 0.249* |
|  |  | (1.130) | (1.042) | (1.041) |  | (0.159) | (0.143) | (0.145) |
| *SHUT* |  | 0.852*** | 1.000*** | 0.992*** |  | 0.127*** | 0.174*** | 0.173*** |
|  |  | (0.201) | (0.184) | (0.184) |  | (0.023) | (0.021) | (0.021) |
| *SEASON* |  | 0.308 | 0.387* | 0.347 |  | 0.075*** | 0.046* | 0.040 |
|  |  | (0.254) | (0.234) | (0.233) |  | (0.028) | (0.026) | (0.026) |
| *DISASTER* |  | -0.203 | 0.264 | 0.328 |  | -0.065 | -0.012 | -0.009 |
|  |  | (0.588) | (0.538) | (0.541) |  | (0.062) | (0.057) | (0.057) |
| *Log(POP)* |  |  |  | 0.254*** |  |  |  | 0.027*** |
|  |  |  |  | (0.066) |  |  |  | (0.008) |
| *Log(INCOMEAVG)* |  |  |  | -0.309 |  |  |  | -0.122* |
|  |  |  |  | (0.610) |  |  |  | (0.072) |
| *EDU* |  |  |  | -0.049*** |  |  |  | -0.008*** |
|  |  |  |  | (0.013) |  |  |  | (0.002) |
| *M/F* |  |  |  | 4.216** |  |  |  | 0.546*** |
|  |  |  |  | (1.668) |  |  |  | (0.197) |
| *Minority* |  |  |  | -2.382*** |  |  |  | -0.347*** |
|  |  |  |  | (0.431) |  |  |  | (0.052) |
| Constant | 6.417*** | 10.491*** | 11.828*** | 9.595 | 2.115*** | -2.708*** | -2.387*** | -1.724** |
|  | (0.518) | (0.762) | (0.737) | (5.883) | (0.099) | (0.100) | (0.097) | (0.698) |
| Industry-by-year | No | No | Yes | Yes | No | No | Yes | Yes |
| fixed effects |  |  |  |  |  |  |  |  |
| Observations | 31,662 | 31,662 | 31,650 | 31,623 | 31,662 | 31,662 | 31,650 | 31,623 |
| Adjusted R^2^ | 0.001 | 0.146 | 0.294 | 0.299 | 0.000 | 0.560 | 0.644 | 0.649 |

## 5.2. Religious Adherence near Headquarters Versus Establishments

To run a horse race between establishment-level and headquarter-level religiosity, we include *Religiosity_EM_* and *Religiosity_HQ_* in the same regressions as those shown in SI Table 10. As with the findings in SI Table 10, the coefficient estimate of *Religiosity_EM_* reported in SI Table 11 is again significantly negative, but that of *Religiosity_HQ_* remains insignificant or marginally significantly positive. In sum, we find little evidence that religiosity local to firm headquarter is significantly related worker safety, which is in contrast to the results on religiosity local to establishment. In Panel B of SI Table 5, we estimate the same sets of OLS regressions but without Winsorizing variables. Coefficient estimates of *Religiosity_HQ_* remain insignificant or positively correlated with injury rate and total case count when no Winsorizing takes place.

### SI Table 11. Religious Adherence near Headquarters versus Establishments

The relationship between the degree of religious affiliation among residents in the county in which an enterprise’s headquarters is located and measures of workplace injury in its establishments.  *TCR* is calculated as “the number of recordable cases (defined as the sum of total number of deaths and all injuries and illnesses that results in days away from work or with job transfer or restriction, and other recordable cases) × 200,000 / Employee hours worked” ^11^. *TC* is the “total number of recordable cases (including deaths and all injuries and illnesses)” as defined above^11^. *Religiosity_EM_* is the proportion of residents acknowledging a religious adherence in a county where the establishment is located^12-14^. *Religiosity_HQ_* is the religiosity measure in a county where the firm’s headquarter is located^12-14^. Detailed definitions of all other covariates are shown in SI Table 1. Standard errors are clustered by establishments and reported underneath coefficient estimates. Asterisks denote the level of p-values: *** *p*<0.01, ** *p*<0.05, **p*<0.10.

| Dependent Variable | *TCR_t+1_* | *Ln(1+TC)_t+1_* |
| --- | --- | --- |
|  | (1) | (2) |
| *Religiosity_EM_* | -1.486*** | -0.196*** |
|  | (0.540) | (0.064) |
| *Religiosity_HQ_* | 0.896 | 0.202* |
|  | (0.829) | (0.104) |
| *Ln(Assets)* | 0.036 | -0.024*** |
|  | (0.049) | (0.006) |
| *LEV* | 1.379*** | 0.225*** |
|  | (0.394) | (0.050) |
| *PPETA* | 2.051*** | 0.123* |
|  | (0.544) | (0.068) |
| *SALETA* | 0.423*** | 0.087*** |
|  | (0.125) | (0.015) |
| *CAPEX_Assets* | 7.169*** | 1.369*** |
|  | (2.311) | (0.297) |
| *MTB* | -0.034 | 0.008 |
|  | (0.078) | (0.011) |
| *FCFTA* | 1.417** | 0.137 |
|  | (0.708) | (0.100) |
| *CASHTA* | 1.396** | -0.127 |
|  | (0.660) | (0.091) |
| *DIVTA* | -3.024 | 0.393 |
|  | (3.947) | (0.554) |
| *Ln(EMP)* | 0.224*** | 0.868*** |
|  | (0.064) | (0.008) |
| *HRS* | -0.004*** | -0.000 |
|  | (0.000) | (0.000) |
| *STRIKE* | 1.464 | 0.251* |
|  | (1.042) | (0.146) |
| *SHUT* | 0.983*** | 0.172*** |
|  | (0.184) | (0.021) |
| *SEASON* | 0.365 | 0.042* |
|  | (0.233) | (0.026) |
| *DISASTER* | 0.295 | -0.014 |
|  | (0.538) | (0.056) |
| *Log(POP)* | 0.253*** | 0.027*** |
|  | (0.066) | (0.008) |
| *Log(INCOMEAVG)* | -0.323 | -0.124* |
|  | (0.610) | (0.071) |
| *EDU* | -0.048*** | -0.008*** |
|  | (0.013) | (0.002) |
| *M/F* | 3.463** | 0.447** |
|  | (1.685) | (0.199) |
| *Minority* | -2.478*** | -0.360*** |
|  | (0.435) | (0.053) |
| Constant | 11.029* | -1.535** |
|  | (5.891) | (0.700) |
| Industry-by-year fixed effects | Yes | Yes |
| Observations | 31,623 | 31,623 |
| Adjusted R^2^ | 0.300 | 0.650 |

## 5.3. Religious Denomination and Workplace Injury

Prior literature shows that Protestants display higher level of risk-aversion than Catholics^13,24^ and greater cooperativeness^25^. Accordingly, we compute the respective number of Protestant and Catholic religious adherents in counties local to the private-sector establishments and we divided those counts by the counties’ total populations(*Protestant* and *Catholic)* in the county where an establishment is located^12-14^. We, then, replicate the regressions in Panel A of SI Table 10, albeit with the rates of Protestant and Catholic adherence, respectively, or collectively, added to replace the focal predictor in the model.

As shown in columns (1) and (2) of SI Table 12, the level of *Protestant_EM_* religious adherence is significantly negatively related to total injury rate *TCR* and the number of total cases *Ln(1+TC)*. In columns (3) and (4), we observe significant positive coefficient estimates on *Catholic_EM_* in both regressions explaining *TCR* and *Ln(1+TC)*. The finding is in line with that in prior research^13^, which shows local Protestant ratio is negatively related to mutual fund return volatilities but local Catholic ratio is positively associated mutual fund return volatilities. As we include both *Protestant_EM_* and *Catholic_EM_* in the same regressions as shown in columns (5) and (6), coefficient estimate on *Protestant_EM_* remains significantly negative, however the coefficient estimate on *Catholic_EM_* is statistically insignificant. This suggests that protestant religiosity improves worker safety, but Catholic religiosity does not. Notably, to the extent that Protestantism is the largest religious group in the U.S., it is not surprising to observe an overall negative relationship between total religious adherence and the injury rate.

### SI Table 12. Religious Denomination and Workplace Injury

Diverse relationships between the degree of affiliation with particular religious denominations and workplace injury. *TCR* is calculated as “the number of recordable cases (defined as the sum of total number of deaths and all injuries and illnesses that results in days away from work or with job transfer or restriction, and other recordable cases) × 200,000 / Employee hours worked” ^11^. *TC* is the “total number of recordable cases (including deaths and all injuries and illnesses)” as defined above^11^. *Protestant_EM_* and *Catholic_EM_* represents a county’s proportion of residents acknowledging a religious adherence to, respectively, the Protestant Christian religion and the Catholic Christian religion^12-14^. Detailed definitions of all other covariates are shown in SI Table 1. Standard errors are clustered by establishments and reported underneath coefficient estimates. Asterisks denote the level of p-values: *** *p*<0.01, ** *p*<0.05, **p*<0.10.

| Dependent Variable | *TCR_t+1_* | *Ln(1+TC)_t+1_* | *TCR_t+1_* | *Ln(1+TC)_t+1_* | *TCR_t+1_* | *Ln(1+TC)_t+1_* |
| --- | --- | --- | --- | --- | --- | --- |
|  | (1) | (2) | (3) | (4) | (5) | (6) |
| *Protestant_EM_* | -1.467*** | -0.180*** |  |  | -1.258*** | -0.146*** |
|  | (0.401) | (0.047) |  |  | (0.435) | (0.051) |
| *Catholic_EM_* |  |  | 1.358*** | 0.184*** | 0.596 | 0.095 |
|  |  |  | (0.506) | (0.061) | (0.547) | (0.066) |
| *Ln(Assets)* | 0.030 | -0.025*** | 0.035 | -0.024*** | 0.032 | -0.024*** |
|  | (0.049) | (0.006) | (0.049) | (0.006) | (0.049) | (0.006) |
| *LEV* | 1.280*** | 0.210*** | 1.303*** | 0.213*** | 1.290*** | 0.211*** |
|  | (0.399) | (0.050) | (0.399) | (0.050) | (0.399) | (0.050) |
| *PPETA* | 2.039*** | 0.128* | 2.021*** | 0.127* | 2.045*** | 0.129* |
|  | (0.543) | (0.068) | (0.544) | (0.068) | (0.543) | (0.068) |
| *SALETA* | 0.405*** | 0.084*** | 0.403*** | 0.083*** | 0.407*** | 0.084*** |
|  | (0.125) | (0.015) | (0.125) | (0.015) | (0.125) | (0.015) |
| *CAPEXTA* | 6.638*** | 1.242*** | 6.806*** | 1.265*** | 6.716*** | 1.254*** |
|  | (2.278) | (0.293) | (2.289) | (0.295) | (2.282) | (0.294) |
| *MTB* | -0.029 | 0.009 | -0.031 | 0.009 | -0.031 | 0.009 |
|  | (0.079) | (0.011) | (0.079) | (0.011) | (0.079) | (0.011) |
| *FCFTA* | 1.184* | 0.110 | 1.145 | 0.105 | 1.182* | 0.110 |
|  | (0.716) | (0.101) | (0.718) | (0.101) | (0.716) | (0.101) |
| *CASHTA* | 1.115 | -0.176* | 1.178* | -0.168* | 1.138* | -0.172* |
|  | (0.680) | (0.092) | (0.682) | (0.092) | (0.680) | (0.092) |
| *DIVTA* | -1.502 | 0.572 | -2.044 | 0.504 | -1.613 | 0.554 |
|  | (4.042) | (0.562) | (4.041) | (0.563) | (4.040) | (0.562) |
| *Ln(EMP)* | 0.235*** | 0.870*** | 0.234*** | 0.869*** | 0.236*** | 0.870*** |
|  | (0.064) | (0.008) | (0.064) | (0.008) | (0.064) | (0.008) |
| *HRS* | -0.004*** | -0.000 | -0.004*** | -0.000 | -0.004*** | -0.000 |
|  | (0.000) | (0.000) | (0.000) | (0.000) | (0.000) | (0.000) |
| *STRIKE* | 1.452 | 0.249* | 1.449 | 0.248* | 1.444 | 0.248* |
|  | (1.046) | (0.146) | (1.043) | (0.146) | (1.047) | (0.146) |
| *SHUT* | 0.954*** | 0.166*** | 0.967*** | 0.167*** | 0.954*** | 0.166*** |
|  | (0.182) | (0.021) | (0.183) | (0.021) | (0.182) | (0.021) |
| *SEASON* | 0.357 | 0.043* | 0.350 | 0.042 | 0.352 | 0.042 |
|  | (0.231) | (0.026) | (0.232) | (0.026) | (0.231) | (0.026) |
| *DISASTER* | 0.316 | -0.014 | 0.357 | -0.009 | 0.332 | -0.012 |
|  | (0.535) | (0.056) | (0.539) | (0.057) | (0.536) | (0.056) |
| *Log(POP)* | 0.157** | 0.015* | 0.194*** | 0.019** | 0.146** | 0.014 |
|  | (0.070) | (0.009) | (0.070) | (0.009) | (0.071) | (0.009) |
| *Log(INCOMEAVG)* | -0.629 | -0.159** | -0.694 | -0.171** | -0.731 | -0.176** |
|  | (0.607) | (0.071) | (0.620) | (0.072) | (0.617) | (0.072) |
| *EDU* | -0.045*** | -0.007*** | -0.043*** | -0.007*** | -0.044*** | -0.007*** |
|  | (0.013) | (0.002) | (0.013) | (0.002) | (0.013) | (0.002) |
| *M/F* | 3.187* | 0.411** | 4.332*** | 0.552*** | 3.373** | 0.440** |
|  | (1.665) | (0.200) | (1.657) | (0.196) | (1.676) | (0.201) |
| *Minority* | -2.432*** | -0.355*** | -2.294*** | -0.337*** | -2.375*** | -0.346*** |
|  | (0.425) | (0.052) | (0.432) | (0.052) | (0.433) | (0.052) |
| Constant | 15.743*** | -0.931 | 13.941** | -1.110 | 16.455*** | -0.818 |
|  | (5.948) | (0.700) | (5.975) | (0.704) | (6.008) | (0.705) |
| Industry-by-year  fixed effects | Yes | Yes | Yes | Yes | Yes | Yes |
| Observations | 31,835 | 31,835 | 31,835 | 31,835 | 31,835 | 31,835 |
| Adjusted R^2^ | 0.299 | 0.649 | 0.299 | 0.649 | 0.299 | 0.649 |

## ***5.4. Religion’s Effect on Injuries in the Presence of Strong Labor Unions***

Our findings suggest that the rate of religious adherence influences worker safety, just as past research indicates that religious adherence increases prosocial behaviors. However, the latter line of inquiry also reports that the effects of religious adherence become muted in the presence of “earthly” authorities that can sanction misbehavior^26^. In the context of our study, labor unions perform this “earthly” role by monitoring working conditions and threatening sanctions—such as work stoppages or law suits—if workplace conditions are unsafe. Thus, we posit that religious adherence will have a stronger effect on worker safety in establishments with weak union power.

To test this conjecture, we partition our full sample into two subgroups, one with strong union power and the other with weak union power. Then, in each subsample, we regressed our measures of workplace injury on local religious adherence and our set of covariates and fixed effects. Two variables proxy for union power in these analyses and are used to divide the subsample. The first is *Union Membership*—the percent of employees in an industry, during a given year, who are members of a union^e.g., see 16^. The second proxy is *Bargaining Agreement*—the percent of employees in an industry-year who are included in a collective bargaining agreement ^e.g., see 16^. Previous research^16^ combines these measures, though we treat them as separate variables that represent union power. We partition our sample based on the sample medians of these two measures in a year. We find that the coefficient estimate on *Religiosity_EM_* is negative and significant at the 1% level only in establishments with low *Union Membership*. In contrast the coefficient estimate on *Religiosity_EM_* is insignificant in establishments with high *Union Membership.* As we turn to columns (3) and (4), two subsamples are created based on whether an establishment has high or low industry *Bargain Agreement,* partitioned by the sample median in a year. We find local religiosity is negatively associated with injury rates only in establishments that have a low *Bargain Agreement.* In contrast, there is little significant relationship between religiosity and injury rates in establishments with high *Bargain Agreement*. The Wald tests of the differences between the coefficient estimates on *Religiosity_EM_* across columns (1) and (2) as well as columns (3) and (4) indicate significant differences in the effects of local religiosity on the injury rates across the two subsamples. These findings are consistent with our conjecture that local religiosity substitutes for the union role in promoting worker safety.

### SI Table 13. **Religion’s Effect on Injuries in the Presence of Strong Labor Unions**

The relationship between the degree of local religiosity and workplace safety conditional on union power. Columns (1) and (2) show the relationship in the subsample where the *Union Membership*-the percent of employees in an industry, during a given year, who are members of a union^e.g., see 16^ is above or equal to (1) or below (2) the sample median. Columns (3) and (4) show the relationship in the subsample where the *Bargain Agreement*-the percent of employees in an industry-year who are included in a collective bargaining agreement ^e.g., see 16^ is above or equal to (3) or below (4) the sample median. *TCR* is calculated as “the number of recordable cases (defined as the sum of total number of deaths and all injuries and illnesses that results in days away from work or with job transfer or restriction, and other recordable cases) × 200,000 / Employee hours worked” ^11^. *Religiosity_EM_* is the proportion of residents acknowledging a religious adherence in a county where the establishment is located^12-14^. Detailed definitions of all other covariates are shown in SI Table 1. Standard errors are clustered by establishments and reported underneath coefficient estimates. Asterisks denote the level of p-values: *** *p*<0.01, ** *p*<0.05, **p*<0.10.

|  | Union Membership | | Bargain Agreement | |
| --- | --- | --- | --- | --- |
|  | High | Low | High | Low |
| Dependent Variable | *TCR_t+1_* | *TCR_t+1_* | *TCR_t+1_* | *TCR_t+1_* |
|  | (1) | (2) | (3) | (4) |
| *Religiosity_EM_* (β1) | -0.300 | -2.772*** | -0.203 | -2.883*** |
|  | (0.826) | (0.616) | (0.827) | (0.617) |
| *Ln(Assets)* | 0.102 | -0.083 | 0.122 | -0.090 |
|  | (0.077) | (0.059) | (0.078) | (0.060) |
| *LEV* | 0.625 | 1.921*** | 0.434 | 1.902*** |
|  | (0.711) | (0.476) | (0.712) | (0.481) |
| *PPETA* | 5.144*** | -2.868*** | 5.100*** | -3.354*** |
|  | (0.790) | (0.714) | (0.770) | (0.722) |
| *SALETA* | 0.107 | 0.784*** | 0.133 | 0.798*** |
|  | (0.176) | (0.156) | (0.174) | (0.158) |
| *CAPEXTA* | -6.407** | 29.875*** | -5.707* | 30.821*** |
|  | (3.254) | (3.067) | (3.225) | (3.097) |
| *MTB* | -0.252 | -0.154* | -0.277* | -0.153* |
|  | (0.154) | (0.086) | (0.154) | (0.088) |
| *FCFTA* | 3.670*** | 0.748 | 3.579*** | 0.844 |
|  | (1.367) | (0.822) | (1.336) | (0.836) |
| *CASHTA* | 3.679*** | -0.699 | 3.526*** | -0.798 |
|  | (1.348) | (0.746) | (1.351) | (0.755) |
| *DIVTA* | -5.996 | -12.095** | -6.788 | -11.495** |
|  | (6.237) | (5.607) | (6.268) | (5.735) |
| *Ln(EMP)* | 0.113 | 0.187** | 0.101 | 0.212*** |
|  | (0.090) | (0.075) | (0.090) | (0.076) |
| *HRS* | -0.005*** | -0.003*** | -0.005*** | -0.003*** |
|  | (0.000) | (0.000) | (0.000) | (0.000) |
| *STRIKE* | 1.181 | 3.999 | 1.255 | 3.999 |
|  | (1.148) | (2.474) | (1.149) | (2.467) |
| *SHUT* | 0.791*** | 0.838*** | 0.860*** | 0.807*** |
|  | (0.292) | (0.233) | (0.294) | (0.228) |
| *SEASON* | 0.896** | 0.170 | 1.006*** | 0.124 |
|  | (0.376) | (0.285) | (0.380) | (0.282) |
| *DISASTER* | -0.041 | 0.572 | 0.131 | 0.438 |
|  | (0.801) | (0.698) | (0.809) | (0.694) |
| *Log(POP)* | 0.216** | 0.351*** | 0.200** | 0.374*** |
|  | (0.101) | (0.079) | (0.100) | (0.081) |
| *Log(INCOMEAVG)* | -0.654 | -0.100 | -0.614 | -0.096 |
|  | (0.999) | (0.656) | (1.001) | (0.661) |
| *EDU* | -0.028 | -0.052*** | -0.028 | -0.053*** |
|  | (0.022) | (0.014) | (0.022) | (0.014) |
| *M/F* | 7.814*** | 1.509 | 7.634*** | 1.560 |
|  | (2.704) | (1.797) | (2.709) | (1.800) |
| *Minority* | -1.724** | -3.163*** | -1.667** | -3.210*** |
|  | (0.709) | (0.463) | (0.705) | (0.466) |
| Constant | 12.291 | 8.787 | 12.230 | 8.456 |
|  | (9.658) | (6.349) | (9.679) | (6.406) |
| Wald test on (β1) | χ2=13.35*** | | χ2=15.71*** | |
|  | (<0.001) | | (<0.001) | |
| Industry-by-year  fixed effects | Yes | Yes | Yes | Yes |
| Observations | 14,814 | 14,789 | 14,804 | 14,799 |
| Adjusted R^2^ | 0.300 | 0.333 | 0.298 | 0.333 |

## 5.5. Cooperation as a Mechanism underlying Religion’s Relationship to Workplace Safety.

To investigate this possible mechanism, we focus on firm-level analysis since data on safety investments are unavailable at the establishment-level. Nevertheless, firm-level safety investments may reflect aggregated spending on safety resulted from individual plant-level managerial decisions that are shaped by local religious beliefs around each establishment. It is challenging to measure the firms’ investments in workplace safety because it involves not only direct expenses, such as replacing aging equipment with safer models, but also expenditures on intangible activities “such as safety training and program, safety incentives, and safety consulting”^27^. We construct two variables to proxy for firm level workplace safety investment. Following past work^7^, we proxy investments in safety using abnormal discretionary expenses per employee (*AbDisExp*), which is estimated based on previous methods^28^. Additionally, for robustness, we also construct an alternatively firm-level safety performance measure based on the ratings from the “MSCI ESG KLD STATS” data set from MSCI ESG Research, which is the largest provider of environmental, social and governance research^29^. This data set presents a binary (i.e., 0/1) summary of “strength” and “concern” on ESG ratings, including health and safety. We take the rating of “Health and Safety Strength” and subtract that of “Health and Safety Concern” to construct the KLD Safety Index, following prior research^3^. A higher index value therefore indicates better safety performance. Assuming a firm’s overall safety performance is a result of corporate investments in tangible and intangible assets strengthening safety, we expect the safety index to serve as a reasonable proxy for safety investment.

To conduct firm-level analysis, we aggregate annual establishment-level religiosity into a firm-level variable called *Religiosity_EM_Aggregated_*, which is defined as the weighted-average value of *Religiosity_EM_* of all establishments of a firm, weighted by establishment employee headcount.

As shown in columns (1) and (3) in SI Table 14, we regress *AbDisExp* and *KLD* *Safety Index*, respectively, on the *Religiosity_EM_Aggregated_* plus a set of firm-level vairables as control. To assess whether headquarter-level religiosity affects safety investments, we include *Religiosity_HQ_* as an independent variable in columns (2) and (5), and both *Religiosity_EM_Aggregated_* and *Religiosity_HQ_* in columns (3) and (6). We follow prior research^30^ and include *Macro Q* and *Cash flow* as control variables explaining *AbDisExp* in the OLS models (1)‒(3). In columns (4)‒(6), where the dependent variable is *KLD Safety Index*, we estimate ordered logit models and include *Ln(Assets)*, *KZ*, *ROA*, *CASHTA*, *MTB*, and *DIVTA* as control variables, following past work^31^. Prior studies have shown that these firm-characteristics are related to KLD ratings^31^. In addition, we include in the model year and firm fixed effects.

We find that *Religiosity_EM_Aggregated_* is significantly and positively related to safety investment proxied by both *AbDisExp* and *KLD Safety Index*, even after controlling for *Religiosity_HQ_*. In contrast, the coefficient estimates on *Religiosity_HQ_* are insignificant except in column (2). As we control for aggregated religiosity at establishment level (see columns (3) and (6)), headquarter-religiosity becomes insignificantly related to *AbDisExp* and *KLD Safety Index*.

### SI Table 14. Cooperation as A Mechanism Underlying Religion’s Relationship to Workplace Safety

In this table, we examine the effect of local religiosity on overall firm level workplace safety investment. Following past work^7^, we proxy investments in safety using abnormal discretionary expenses per employee (*AbDisExp*), which is estimated based on previous methods^28^. *KLD Safety Index* is computed by taking the rating of “Health and Safety Strength” and subtracting that of “Health and Safety Concern” in the “MSCI ESG KLD STATS” data set from MSCI ESG Research^29^, following prior research^3^. A higher index value therefore indicates better safety performance. *Religiosity_EM_Aggregated_* is the annual weighted-average value of *Religiosity_EM_* of all establishments for a firm, weighted by *EMP* in each establishment, where *Religiosity_EM_* is the proportion of residents acknowledging a religious adherence in a county where the establishment is located^12-14^. *Religiosity_HQ_* is the religiosity measure in a county where the firm’s headquarter is located^12-14^. Detailed definitions of all other covariates are shown in SI Table 1. Standard errors are clustered by establishments and reported underneath coefficient estimates. Asterisks denote the level of p-values: *** *p*<0.01, ** *p*<0.05, **p*<0.10.

| Dependent Variable | *AbDisExp_t_* | *AbDisExp_t_* | *AbDisExp_t_* | *KLD Safety Index_t_* | *KLD Safety Index_t_* | *KLD Safety Index_t_* |
| --- | --- | --- | --- | --- | --- | --- |
|  | (1) | (2) | (3) | (4) | (5) | (6) |
| *Religiosity_EM_Aggregated_* | 20.931* |  | 19.828* | 4.406*** |  | 4.438*** |
|  | (10.734) |  | (10.886) | (1.510) |  | (1.484) |
| *Religiosity_HQ_* |  | 74.900* | 68.949 |  | -2.711 | -3.703 |
|  |  | (42.733) | (42.702) |  | (11.758) | (11.509) |
| *Macro Q* | 0.008 | 0.008 | 0.008 |  |  |  |
|  | (0.006) | (0.006) | (0.006) |  |  |  |
| *Cash flow* | -0.203* | -0.204* | -0.203* |  |  |  |
|  | (0.119) | (0.119) | (0.118) |  |  |  |
| *Ln(Assets)* |  |  |  | -0.978** | -0.945** | -0.963** |
|  |  |  |  | (0.430) | (0.440) | (0.435) |
| *KZ* |  |  |  | -0.001 | -0.001 | -0.001 |
|  |  |  |  | (0.001) | (0.001) | (0.001) |
| *ROA* |  |  |  | 1.132 | 0.851 | 1.149 |
|  |  |  |  | (1.570) | (1.579) | (1.568) |
| *CASHTA* |  |  |  | 0.222 | 0.036 | 0.226 |
|  |  |  |  | (1.579) | (1.627) | (1.587) |
| *MTB* |  |  |  | -0.352 | -0.308 | -0.342 |
|  |  |  |  | (0.221) | (0.221) | (0.222) |
| *DIVTA* |  |  |  | -6.027 | -5.537 | -6.149 |
|  |  |  |  | (11.258) | (11.212) | (11.341) |
| Constant | -24.946*** | -53.564** | -60.701*** | -1.092 | -5.727 | -3.499 |
|  | (5.769) | (22.655) | (22.899) | (2.996) | (7.979) | (7.945) |
| Firm and year fixed effects | Yes | Yes | Yes | Yes | Yes | Yes |
| Observations | 5,966 | 5,890 | 5,890 | 3,369 | 3,344 | 3,344 |
| Adjusted R^2^ or Pseudo R^2^ | 0.808 | 0.808 | 0.808 | 0.574 | 0.568 | 0.57 |

## 5.6. Self-Control and ***Risk Aversion as Mechanisms underlying Religion’s Relationship to Workplace Safety***

We study how the relationship between the degree of affiliation with particular religious denominations and workplace injury is affected by individual traits related to being cautious or complying. We proxy such a personality trait using local auto accident rate. Lack of caution or incompliance with traffic rules naturally leads to a high auto accident rate. If religiosity indeed reduces injury rate via improving workers’ safety compliance and caution at job, we expect the effect of religiosity should be stronger (i.e., more negative) for workers who tend to be careless and incompliant with rules, e.g., those with a higher auto accident rate.

We collect data on state-level auto accident and fatality rates from the NHTSA^9^.*Auto Accident Rate* is the state-level “fatality rate per 100 million vehicle miles traveled”, as defined by Unites States Department of Transportation^9^. As shown in SI Table 15, we partition our full sample into two subgroups—those above and those below the median auto accident rate in the state where an establishment is located, then estimate OLS regressions in each subsample. In columns (1) and (2), where the dependent variable is *TCR*, the coefficient estimate on *Religiosity_EM_* is significantly negative in the group with a high *Auto Accident Rate*, but insignificant in the group with a low *Auto Accident Rate*. As we turn to columns (3) and (4), where the dependent variable is *Ln(1+TC)*, we once again find that *Religiosity_EM_* is negatively associated with total cases count only in the subsample with high *Auto Accident Rate.*

### SI Table 15. Self-Control and **Risk Aversion as Mechanisms Underlying Religion’s Relationship to Workplace Safety**

In this table, we present the results of the relationship between religiosity and workplace safety conditional on auto accident rate in the state where the establishment is located. *TCR* is calculated as “the number of recordable cases (defined as the sum of total number of deaths and all injuries and illnesses that results in days away from work or with job transfer or restriction, and other recordable cases) × 200,000 / Employee hours worked” ^11^. *TC* is the “total number of recordable cases (including deaths and all injuries and illnesses)” as defined above^11^. *Religiosity_EM_* is the proportion of residents acknowledging a religious adherence in a county where the establishment is located^12-14^. In columns (1)-(2) and (3)-(4), we divide the sample into two groups: High vs. Low *Auto Accident Rate* – those above and those below the median auto accident rate in the state where an establishment is located, then estimate OLS regressions in each subsample *Auto Accident Rate* is the state-level “fatality rate per 100 million vehicle miles traveled”, as defined by Unites States Department of Transportation^9^. Detailed definitions of all other covariates are shown in SI Table 1. Standard errors are clustered by establishments and reported underneath coefficient estimates. Asterisks denote the level of p-values: *** *p*<0.01, ** *p*<0.05, **p*<0.10.

|  | Auto Accident Rate | | Auto Accident Rate | |
| --- | --- | --- | --- | --- |
|  | High | Low | High | Low |
| Dependent Variable | *TCR_t+1_* | *TCR_t+1_* | *Ln(1+TC)_t+1_* | *Ln(1+TC)_t+1_* |
|  |  |  |  |  |
|  | (1) | (2) | (3) | (4) |
| *Religiosity_EM_ (β1)* | -1.700** | -0.989 | -0.253*** | -0.061 |
|  | (0.729) | (0.719) | (0.086) | (0.086) |
| *Ln(Assets)* | 0.007 | 0.020 | -0.036*** | -0.019** |
|  | (0.074) | (0.060) | (0.008) | (0.008) |
| *LEV* | 0.936 | 1.524*** | 0.105 | 0.280*** |
|  | (0.616) | (0.505) | (0.070) | (0.068) |
| *PPETA* | 2.219*** | 2.169*** | 0.072 | 0.241*** |
|  | (0.778) | (0.725) | (0.096) | (0.090) |
| *SALETA* | 0.679*** | 0.111 | 0.098*** | 0.065*** |
|  | (0.179) | (0.161) | (0.021) | (0.020) |
| *CAPEX_Assets* | 7.853** | 5.010 | 1.632*** | 0.757* |
|  | (3.337) | (3.091) | (0.425) | (0.392) |
| *MTB* | 0.124 | -0.152 | 0.037** | -0.010 |
|  | (0.129) | (0.095) | (0.017) | (0.013) |
| *FCFTA* | 2.714** | 0.415 | 0.326** | -0.011 |
|  | (1.252) | (0.870) | (0.162) | (0.128) |
| *CASHTA* | 1.345 | 1.052 | -0.173 | -0.135 |
|  | (1.204) | (0.762) | (0.144) | (0.114) |
| *DIVTA* | -1.771 | -3.095 | -0.332 | 1.016 |
|  | (5.849) | (5.268) | (0.783) | (0.761) |
| *Ln(EMP)* | 0.194** | 0.262*** | 0.880*** | 0.860*** |
|  | (0.098) | (0.076) | (0.011) | (0.011) |
| *HRS* | -0.004*** | -0.004*** | -0.000 | 0.000 |
|  | (0.000) | (0.000) | (0.000) | (0.000) |
| *STRIKE* | 0.738 | 1.904 | 0.394 | 0.150 |
|  | (1.759) | (1.231) | (0.294) | (0.149) |
| *SHUT* | 1.222*** | 0.766*** | 0.186*** | 0.143*** |
|  | (0.288) | (0.226) | (0.030) | (0.028) |
| *SEASON* | 0.484 | 0.286 | 0.043 | 0.054 |
|  | (0.331) | (0.317) | (0.036) | (0.035) |
| *DISASTER* | 0.640 | -0.226 | 0.002 | 0.057 |
|  | (0.611) | (1.086) | (0.064) | (0.115) |
| *Log(POP)* | 0.205** | 0.209** | 0.015 | 0.020* |
|  | (0.102) | (0.091) | (0.012) | (0.011) |
| *Log(INCOMEAVG)* | -0.776 | -0.773 | -0.227** | -0.119 |
|  | (0.912) | (0.892) | (0.101) | (0.105) |
| *EDU* | -0.039** | -0.039* | -0.005** | -0.009*** |
|  | (0.017) | (0.020) | (0.002) | (0.002) |
| *M/F* | 0.041 | 6.169** | 0.162 | 0.557* |
|  | (2.124) | (2.406) | (0.258) | (0.286) |
| *Minority* | -3.341*** | -1.407** | -0.453*** | -0.201*** |
|  | (0.605) | (0.654) | (0.071) | (0.076) |
| Constant | 20.509** | 12.727 | 0.105 | -1.662 |
|  | (8.736) | (8.687) | (0.973) | (1.050) |
| Industry-by-year fixed effects | Yes | Yes | Yes | Yes |
| Observations | 16,094 | 15,741 | 16,094 | 15,741 |
| Adjusted R^2^ | 0.273 | 0.33 | 0.647 | 0.655 |

# 6. Addressing Endogeneity via Instrumental Variable Analyses

The above results suggest that local religious belief has a significant positive effect on workplace safety. However, it is possible that both injury rates and local religiosity are affected by unobservable characteristics, i.e., the omitted variable problem. Alternatively, it is possible that religious counties are attracted to firms with better workplace safety, i.e., the reverse causality problem.

We estimate the effect of religiosity with an instrumental variable (IV) approach, following existing literature on religiosity^12,14^, in two-stage least squares (2SLS) models to mitigate above mentioned concerns. Specifically, we the level of religiosity (*Religiosity_EM_,_t-3_*) and three-years lagged level of population (*Log(POP)_t-3_*) in a county as instruments for *Religiosity_EM_* ^12,14^. *We adopt this previously used approach while noting a peculiar feature of it: the lagged values of our instrument result largely from the linear interpolation of data values, thus raising the possibility that the instrument’s correlation with the endogenous regressor is an artifact of data imputation procedures*. Past research provides some evidence suggesting that the instrument remains valid even absent linear interpolation^12^. Historical religiosity and county population are likely to be correlated with the current level of *Religiosity_EM_.* For example, religiosity is persistent over time, so we expect historical religiosity to be positively associated with present religiosity (the relevance condition). Nevertheless, the instrumental variables do not have an obvious effect on injury rates except through their effects on religiosity (the exclusion condition). Therefore, lagged degree of religious adherence and county population are likely to be valid instruments, as they satisfy both the relevance condition and the exclusion condition.

We include the same vector of covariates as in model (4) of SI Table 10 in the 2SLS regressions. Results are reported in Panel A of SI Table 16. In column (1), we report the result of the first stage model where *Religiosity_EM_* is the dependent variable. The instrument variables, *Religiosity_EM_,_t-3_* and *Log(POP)_t-3_* are positively and negatively related to *Religiosity_EM_,_t_*, respectively, and both coefficient estimates are highly significant, implying that both instruments are relevant. Second-stage results where *TCR* and *TC* are dependent variables are reported in columns (2)–(3), respectively. To assess the relevancy and validity of our instruments, we perform three post-estimation tests. First, we test whether our model is under-identified using Kleibergen-Paap rk LM tests. The tests are highly significant (p-value<1%) for both 2SLS regressions, indicating that the model is identified, i.e., the instruments are relevant. Second, we perform the weak-identification tests (first stage F-tests) and the test results are again highly significant (p-value<1%) in both regressions, indicating that our instruments are strong. Lastly, the p-values of Hansen’s J test are between 41% and 82%, which indicate that we cannot reject the joint null hypothesis that our instrument variables are exogenous. These results suggest our instrument variables are valid, as they satisfy both the relevance criterion and the exclusion criterion. In columns (2)–(3), the coefficient estimates of *Religiosity_EM_* are negative and significant at the 5% level, suggesting that the positive effect of religious adherence on establishment-level worker safety is robust after controlling for endogeneity.

In Panel B, we employ an alternative instrumental variable based on natural disaster events. Natural disaster data is obtained from the “Spatial Hazard Events and Losses Database” (SHELDUS)^17^. This database provides county-level “economic losses, injuries, and fatalities” information associated with 18 types of natural disaster hazard events. The 18 types of natural disasters include “hurricanes/tropical storms, earthquakes, volcanic eruptions, tsunamis, tornadoes, severe thunderstorms, floods, landslides, wildfires, avalanches, coastal hazards, draughts, fogs, hails, heats, lightning events, wind hazards and winter weather hazards”^17^. For each county-year, we construct a deadly disaster indicator variable (*Deadly Disaster*) that takes the value of one if there is at least one death due to any of these natural disaster events, and zero otherwise. We replace the lagged level of population with lagged deadly disaster dummy (*Deadly Disaster_t-3_*). Prior literature suggests that religion conversion rates increase after natural disasters^32,33^. Therefore, the *Deadly Disaster* dummy satisfies the relevance condition. We focus on deadly disasters because prior literature argues that death caused by natural disasters have much stronger impact on religious beliefs and practices than economic losses^34^. On the other hand, there is no obvious reason for natural disaster to be directly related to future workers’ injury rate other than through its effect on religiosity. Therefore, it is likely to satisfy the exclusion condition.

The results are similar to those in Panel A of SI Table 10. Both instruments, *Religiosity_EM_,_t-3_* and *Deadly Disaster_t-3_* are positively and significantly related to *Religiosity_EM_,_t_* in the first stage regression. Our post-estimation tests again suggest our instrument variables satisfy both the relevance criterion and the exclusion criterion. In columns (2)–(3), the coefficient estimates of *Religiosity_EM_,_t_* are again negative and significant at the 5% level in the second stage regressions.

In SI Table 17, we estimate the same sets of 2SLS regressions but using *Religiosity_HQ_* as the independent variable. As with the OLS results, religiosity local to firm headquarter remains insignificantly related to injury rate even after correcting for the endogeneity issue. Overall, the results in this subsection lend support to the hypothesis that local religiosity improves worker safety.

### SI Table 16 – Instrumental Variable Analysis of Community Religiosity and Workplace Safety

This table presents the instrumental variable (IV) regression results estimating the effect of local religiosity on workplace injury rates. *TCR* is calculated as “the number of recordable cases (defined as the sum of total number of deaths and all injuries and illnesses that results in days away from work or with job transfer or restriction, and other recordable cases) × 200,000 / Employee hours worked” ^11^. *TC* is the “total number of recordable cases (including deaths and all injuries and illnesses)” as defined above^11^. *Religiosity_EM_* is the proportion of residents acknowledging a religious adherence in a county where the establishment is located^12-14^. In Panel A, , we use the level of religiosity (*Religiosity_EM_,_t-3_*) and three-years lagged level of population (*Log(POP)_t-3_*) in a county as instruments for *Religiosity_EM_*^12,14^. In Panel B, we use three-years lagged level of religiosity (*Religiosity_EMt-3_*) and a binary variable that equals one if there is death resulted from natural disaster (*Deadly Disaster_t-3_*)^17^ in the county three years prior as instruments for *Religiosity_EM_*. Detailed definitions of all other covariates are shown in SI Table 1. Standard errors are clustered by establishments and reported underneath coefficient estimates. Asterisks denote the level of p-values: *** *p*<0.01, ** *p*<0.05, **p*<0.10.

**Panel A: Lagged religiosity and population as IVs**

|  | First stage | Second stage | |
| --- | --- | --- | --- |
| Dependent Variable | *Religiosity_EM,t_* | *TCR_t+1_* | *Ln(1+TC)_t+1_* |
|  | (1) | (2) | (3) |
| *Religiosity_EM_,_t_* |  | -1.299** | -0.174*** |
|  |  | (0.539) | (0.065) |
| *Religiosity_EM,t-3_* | 0.971*** |  |  |
|  | (0.002) |  |  |
| *Log(POP)_t-3_* | -0.039*** |  |  |
|  | (0.007) |  |  |
| *Ln(Assets)* | -0.000 | 0.026 | -0.025*** |
|  | (0.000) | (0.049) | (0.006) |
| *LEV* | 0.001 | 1.263*** | 0.208*** |
|  | (0.001) | (0.397) | (0.050) |
| *PPETA* | 0.002 | 1.998*** | 0.124* |
|  | (0.002) | (0.539) | (0.068) |
| *SALETA* | 0.000 | 0.373*** | 0.080*** |
|  | (0.000) | (0.123) | (0.015) |
| *CAPEXTA* | -0.000 | 6.459*** | 1.217*** |
|  | (0.007) | (2.266) | (0.291) |
| *MTB* | -0.001** | -0.032 | 0.009 |
|  | (0.000) | (0.079) | (0.011) |
| *FCFTA* | 0.006*** | 1.198* | 0.113 |
|  | (0.002) | (0.711) | (0.100) |
| *CASHTA* | 0.002 | 1.115* | -0.177* |
|  | (0.002) | (0.677) | (0.091) |
| *DIVTA* | -0.002 | -1.794 | 0.539 |
|  | (0.013) | (4.013) | (0.560) |
| *Ln(EMP)* | 0.000 | 0.233*** | 0.869*** |
|  | (0.000) | (0.064) | (0.008) |
| *HRS* | 0.000 | -0.004*** | -0.000 |
|  | (0.000) | (0.000) | (0.000) |
| *STRIKE* | -0.003 | 1.489 | 0.253* |
|  | (0.002) | (1.033) | (0.144) |
| *SHUT* | -0.000 | 0.954*** | 0.165*** |
|  | (0.000) | (0.182) | (0.021) |
| *SEASON* | 0.001 | 0.374 | 0.045* |
|  | (0.001) | (0.230) | (0.025) |
| *DISASTER* | -0.003* | 0.271 | -0.021 |
|  | (0.002) | (0.535) | (0.056) |
| *Log(POP)* | 0.039*** | 0.249*** | 0.027*** |
|  | (0.007) | (0.065) | (0.008) |
| *Log(INCOMEAVG)* | -0.005** | -0.400 | -0.131* |
|  | (0.002) | (0.603) | (0.071) |
| *EDU* | 0.000*** | -0.046*** | -0.007*** |
|  | (0.000) | (0.013) | (0.002) |
| *M/F* | -0.004 | 3.574** | 0.451** |
|  | (0.008) | (1.672) | (0.197) |
| *Minority* | 0.015*** | -2.509*** | -0.366*** |
|  | (0.002) | (0.429) | (0.052) |
| Constant | 0.049** | 17.484*** | -0.927 |
|  | (0.022) | (6.216) | (0.747) |
| *Under-identification test* |  |  |  |
| P value of Kleibergen-Paap rk LM tests | | 0.000*** | 0.000*** |
| *Weak-identification test* |  |  |  |
| P value of first stage F-test |  | 0.000*** | 0.000*** |
| *Over-identification test* |  |  |  |
| P value of Hansen J-test |  | 0.4130 | 0.8206 |
| Industry-by-year fixed effects | Yes | Yes | Yes |
| Observations | 31,811 | 31,811 | 31,811 |
| Adjusted R^2^ | 0.973 | 0.308 | 0.653 |

**Panel B: Lagged religiosity and deadly natural disaster dummy as IVs**

|  | First stage | Second stage | |
| --- | --- | --- | --- |
| Dependent Variable | *Religiosity_EM,t_* | *TCR_t+1_* | *Ln(1+TC)_t+1_* |
|  | (1) | (2) | (3) |
| *Religiosity_EM_,_t_* |  | -1.297** | -0.174*** |
|  |  | (0.539) | (0.065) |
| *Religiosity_EM,t-3_* | 0.969*** |  |  |
|  | (0.002) |  |  |
| *Deadly Disaster_t-3_* | 0.001*** |  |  |
|  | (0.000) |  |  |
| *Ln(Assets)* | -0.000* | 0.026 | -0.025*** |
|  | (0.000) | (0.049) | (0.006) |
| *LEV* | 0.001 | 1.263*** | 0.208*** |
|  | (0.001) | (0.397) | (0.050) |
| *PPETA* | 0.002 | 1.998*** | 0.124* |
|  | (0.002) | (0.539) | (0.068) |
| *SALETA* | 0.000 | 0.373*** | 0.080*** |
|  | (0.000) | (0.123) | (0.015) |
| *CAPEXTA* | 0.001 | 6.459*** | 1.217*** |
|  | (0.007) | (2.266) | (0.291) |
| *MTB* | -0.001*** | -0.032 | 0.009 |
|  | (0.000) | (0.079) | (0.011) |
| *FCFTA* | 0.007*** | 1.198* | 0.113 |
|  | (0.002) | (0.711) | (0.100) |
| *CASHTA* | 0.002 | 1.115* | -0.177* |
|  | (0.002) | (0.677) | (0.091) |
| *DIVTA* | -0.002 | -1.795 | 0.539 |
|  | (0.013) | (4.013) | (0.560) |
| *Ln(EMP)* | 0.000 | 0.233*** | 0.869*** |
|  | (0.000) | (0.064) | (0.008) |
| *HRS* | 0.000 | -0.004*** | -0.000 |
|  | (0.000) | (0.000) | (0.000) |
| *STRIKE* | -0.003 | 1.489 | 0.253* |
|  | (0.002) | (1.033) | (0.144) |
| *SHUT* | -0.000 | 0.954*** | 0.165*** |
|  | (0.000) | (0.182) | (0.021) |
| *SEASON* | 0.001 | 0.374 | 0.045* |
|  | (0.001) | (0.230) | (0.025) |
| *DISASTER* | -0.003* | 0.271 | -0.021 |
|  | (0.002) | (0.535) | (0.056) |
| *Log(POP)* | -0.001*** | 0.249*** | 0.027*** |
|  | (0.000) | (0.065) | (0.008) |
| *Log(INCOMEAVG)* | -0.006** | -0.400 | -0.131* |
|  | (0.002) | (0.603) | (0.071) |
| *EDU* | 0.000*** | -0.046*** | -0.007*** |
|  | (0.000) | (0.013) | (0.002) |
| *M/F* | 0.005 | 3.576** | 0.451** |
|  | (0.007) | (1.672) | (0.197) |
| *Minority* | 0.015*** | -2.509*** | -0.366*** |
|  | (0.002) | (0.429) | (0.052) |
| Constant | 0.047** | 17.481*** | -0.927 |
|  | (0.022) | (6.216) | (0.747) |
| *Under-identification test* |  |  |  |
| P value of Kleibergen-Paap rk LM tests | | 0.000*** | 0.000*** |
| *Weak-identification test* |  |  |  |
| P value of first stage F-test |  | 0.000*** | 0.000*** |
| *Over-identification test* |  |  |  |
| P value of Hansen J-test |  | 0.8877 | 0.5729 |
| Industry-by-year fixed effects | Yes | Yes | Yes |
| Observations | 31,811 | 31,811 | 31,811 |
| Adjusted R^2^ | 0.973 | 0.299 | 0.649 |

**SI Table 17. Instrumental Variable Analysis of Religious Adherence near Headquarters and Workplace Safety**

This table presents the instrumental variable (IV) regression results estimating the effect of religiosity in a county where the firm’s headquarter is located on rate of worker injuries. *TCR* is calculated as “the number of recordable cases (defined as the sum of total number of deaths and all injuries and illnesses that results in days away from work or with job transfer or restriction, and other recordable cases) × 200,000 / Employee hours worked” ^11^. *TC* is the “total number of recordable cases (including deaths and all injuries and illnesses)” as defined above^11^. *Religiosity_HQ_* is the religiosity measure in a county where the firm’s headquarter is located^12-14^. In Panel A, , we use the level of religiosity (*Religiosity_HQ_,_t-3_*) and three-years lagged level of population (*Log(POP_HQ_)_t-3_*) in a county as instruments for *Religiosity_HQ_*^12,14^. In Panel B, we use three-years lagged level of religiosity (*Religiosity_HQt-3_*) and a dummy variable that equals one if there is death resulted from natural disaster (*Deadly Disaster_t-3_*)^17^ in the county three years prior as instruments for *Religiosity_HQ_*. Detailed definitions of all other covariates are shown in SI Table 1. Standard errors are clustered by establishments and reported underneath coefficient estimates. Asterisks denote the level of p-values: *** *p*<0.01, ** *p*<0.05, **p*<0.10.

**Panel A: Lagged religiosity and population as IVs**

|  | First stage | Second stage | |
| --- | --- | --- | --- |
| Dependent Variable | *Religiosity_HQ,t_* | *TCR_t+1_* | *Ln(1+TC)_t+1_* |
|  | (1) | (2) | (3) |
| *Religiosity_HQ_,_t_* |  | 0.224 | 0.122 |
|  |  | (0.803) | (0.104) |
| *Religiosity_HQ,t-3_* | 0.948*** |  |  |
|  | (0.002) |  |  |
| *Log(POP_HQ_)_t-3_* | 0.000 |  |  |
|  | (0.000) |  |  |
| *Ln(Assets)* | -0.000* | 0.040 | -0.024*** |
|  | (0.000) | (0.049) | (0.006) |
| *LEV* | 0.003*** | 1.374*** | 0.225*** |
|  | (0.001) | (0.392) | (0.050) |
| *PPETA* | 0.015*** | 2.034*** | 0.121* |
|  | (0.002) | (0.542) | (0.068) |
| *SALETA* | -0.000 | 0.420*** | 0.086*** |
|  | (0.000) | (0.124) | (0.015) |
| *CAPEXTA* | -0.051*** | 7.187*** | 1.371*** |
|  | (0.006) | (2.301) | (0.296) |
| *MTB* | -0.000 | -0.035 | 0.008 |
|  | (0.000) | (0.078) | (0.011) |
| *FCFTA* | -0.005** | 1.398** | 0.136 |
|  | (0.002) | (0.707) | (0.099) |
| *CASHTA* | 0.002 | 1.335** | -0.135 |
|  | (0.002) | (0.659) | (0.090) |
| *DIVTA* | -0.002 | -2.973 | 0.385 |
|  | (0.012) | (3.918) | (0.550) |
| *Ln(EMP)* | -0.000 | 0.222*** | 0.868*** |
|  | (0.000) | (0.064) | (0.008) |
| *HRS* | -0.000*** | -0.004*** | -0.000 |
|  | (0.000) | (0.000) | (0.000) |
| *STRIKE* | -0.001 | 1.446 | 0.248* |
|  | (0.002) | (1.034) | (0.144) |
| *SHUT* | -0.000 | 0.988*** | 0.173*** |
|  | (0.000) | (0.183) | (0.021) |
| *SEASON* | -0.000 | 0.345 | 0.040 |
|  | (0.001) | (0.231) | (0.025) |
| *DISASTER* | 0.002 | 0.326 | -0.010 |
|  | (0.001) | (0.537) | (0.056) |
| *Log(POP)* | -0.000 | 0.254*** | 0.027*** |
|  | (0.000) | (0.066) | (0.008) |
| *Log(INCOMEAVG)* | -0.000 | -0.310 | -0.123* |
|  | (0.001) | (0.606) | (0.071) |
| *EDU* | -0.000 | -0.049*** | -0.008*** |
|  | (0.000) | (0.013) | (0.002) |
| *M/F* | 0.013*** | 4.213** | 0.545*** |
|  | (0.004) | (1.657) | (0.195) |
| *Minority* | 0.004*** | -2.392*** | -0.348*** |
|  | (0.001) | (0.429) | (0.052) |
| Constant | 0.002 | 14.903** | -1.297* |
|  | (0.014) | (6.244) | (0.748) |
| *Under-identification test* |  |  |  |
| P value of Kleibergen-Paap rk LM tests | | 0.000*** | 0.000*** |
| *Weak-identification test* |  |  |  |
| P value of first stage F-test |  | 0.000*** | 0.000*** |
| *Over-identification test* |  |  |  |
| P value of Hansen J-test |  | 0.993 | 0.539 |
| Industry-by-year fixed effects | Yes | Yes | Yes |
| Observations | 31,619 | 31,619 | 31,619 |
| Adjusted R^2^ | 0.969 | 0.309 | 0.654 |

**Panel B: Lagged religiosity and deadly natural disaster dummy as IVs**

|  | First stage | Second stage | |
| --- | --- | --- | --- |
| Dependent Variable | *Religiosity_EM,t_* | *TCR_t+1_* | *Ln(1+TC)_t+1_* |
|  | (1) | (2) | (3) |
| *Religiosity_HQ_,_t_* |  | 0.220 | 0.122 |
|  |  | (0.803) | (0.104) |
| *Religiosity_HQ,t-3_* | 0.948*** |  |  |
|  | (0.002) |  |  |
| *Deadly Disaster_HQ,t-3_* | 0.001*** |  |  |
|  | (0.000) |  |  |
| *Ln(Assets)* | -0.000* | 0.040 | -0.024*** |
|  | (0.000) | (0.049) | (0.006) |
| *LEV* | 0.003*** | 1.374*** | 0.225*** |
|  | (0.001) | (0.392) | (0.050) |
| *PPETA* | 0.015*** | 2.034*** | 0.121* |
|  | (0.002) | (0.542) | (0.068) |
| *SALETA* | -0.000 | 0.420*** | 0.086*** |
|  | (0.000) | (0.124) | (0.015) |
| *CAPEXTA* | -0.052*** | 7.186*** | 1.371*** |
|  | (0.006) | (2.301) | (0.296) |
| *MTB* | -0.000 | -0.035 | 0.008 |
|  | (0.000) | (0.078) | (0.011) |
| *FCFTA* | -0.005** | 1.399** | 0.136 |
|  | (0.002) | (0.707) | (0.099) |
| *CASHTA* | 0.003 | 1.334** | -0.135 |
|  | (0.002) | (0.659) | (0.090) |
| *DIVTA* | -0.001 | -2.971 | 0.384 |
|  | (0.012) | (3.918) | (0.550) |
| *Ln(EMP)* | -0.000 | 0.222*** | 0.868*** |
|  | (0.000) | (0.064) | (0.008) |
| *HRS* | -0.000*** | -0.004*** | -0.000 |
|  | (0.000) | (0.000) | (0.000) |
| *STRIKE* | -0.001 | 1.446 | 0.248* |
|  | (0.002) | (1.034) | (0.144) |
| *SHUT* | -0.000 | 0.988*** | 0.173*** |
|  | (0.000) | (0.183) | (0.021) |
| *SEASON* | -0.000 | 0.345 | 0.040 |
|  | (0.001) | (0.231) | (0.025) |
| *DISASTER* | 0.002 | 0.326 | -0.010 |
|  | (0.001) | (0.537) | (0.056) |
| *Log(POP)* | -0.000 | 0.254*** | 0.027*** |
|  | (0.000) | (0.066) | (0.008) |
| *Log(INCOMEAVG)* | -0.000 | -0.310 | -0.123* |
|  | (0.001) | (0.606) | (0.071) |
| *EDU* | -0.000 | -0.049*** | -0.008*** |
|  | (0.000) | (0.013) | (0.002) |
| *M/F* | 0.013*** | 4.212** | 0.545*** |
|  | (0.004) | (1.657) | (0.195) |
| *Minority* | 0.004*** | -2.392*** | -0.348*** |
|  | (0.001) | (0.429) | (0.052) |
| Constant | 0.005 | 14.907** | -1.297* |
|  | (0.014) | (6.244) | (0.748) |
| *Under-identification test* |  |  |  |
| P value of Kleibergen-Paap rk LM tests | | 0.000*** | 0.000*** |
| *Weak-identification test* |  |  |  |
| P value of first stage F-test |  | 0.000*** | 0.000*** |
| *Over-identification test* |  |  |  |
| P value of Hansen J-test |  | 0.138 | 0.038** |
| Industry-by-year fixed effects | Yes | Yes | Yes |
| Observations | 31,619 | 31,619 | 31,619 |
| Adjusted R^2^ | 0.969 | 0.309 | 0.654 |

**Supplementary Information References**

1 Administration, O. S. a. H. Vol. Federal Register #: 66:5916-6135 (ed Occupational Safety and Health Administration) (2001).

2 Amin, M. R., Kim, I. & Lee, S. Local religiosity, workplace safety, and firm value. *Journal of Corporate Finance* **70**, 102093, doi:<https://doi.org/10.1016/j.jcorpfin.2021.102093> (2021).

3 Bradley, D., Mao, C. X. & Zhang, C. *Do corporate taxes affect workplace safety?* (Catalogue No. 18-036, 2018).

4 Regulations, C. o. F. in *1904* (United States).

5 OSHA. *2002-2011 ODI Data*, <<https://www.osha.gov/odi/DataDictionary2002-2011.txt>>

6 Cohn, J. B. & Wardlaw, M. I. Financing constraints and workplace safety. *Journal of Finance* **71**, 2917-2058 (2016).

7 Caskey, J. & Ozel, N. B. Earnings expectations and employee safety. *Journal of Accounting and Economics* **63**, 121-141 (2017).

8 Grammich, C. *et al.* (Association of Religion Data Archives, 2018).

9 Administration, N. H. T. S. *FARS Data Tables*, <<https://www-fars.nhtsa.dot.gov/States/StatesCrashesAndAllVictims.aspx>>

10 *Union Membership, Coverage, and Earnings from the CPS*, <<http://www.unionstats.com/>> (

11 Statistics, U. S. B. o. L. *How To Compute a Firm's Incidence Rate for Safety Management*, <<https://www.bls.gov/iif/osheval.htm>>

12 Hilary, G. & Hui, K. W. Does religion matter in corporate decision making in America? *Journal of Financial Economics* **93**, 455-473 (2009).

13 Shu, T., Sulaeman, J. & Yeung, P. E. Local religious beliefs and mutual fund risk-taking behaviors. *Management Science* **58**, 1779-1796 (2012).

14 Jiang, F., Kose, J. C., Li, W. & Qian, Y. Earthly reward to the religious: Religiosity and the cost of public and private debt. *Journal of Financial and Quantitative Analysis* **53**, 2131-2160 (2018).

15 Kaplan, S. N. & Zingales, L. Do investment-cash flow sensitivities provide useful measures of financing constraints? *The quarterly journal of economics* **112**, 169-215 (1997).

16 Falato, A. & Liang, N. Do Creditor Rights Increase Employment Risk? Evidence from Loan Covenants. *The Journal of Finance* **71**, 2545-2590, doi:<https://doi.org/10.1111/jofi.12435> (2016).

17 CEMHS. *Spatial Hazard Events and Losses Database for the United States*, <<https://cemhs.asu.edu/sheldus>>

18 Norenzayan, A. *et al.* The Cultural Evolution of Prosocial Religions. *Behavioral and Brain Sciences* **39**, 1-19 (2016).

19 Chantziaras, A., Dedoulis, E., Grougiou, V. & Leventis, S. The impact of religiosity and corruption on CSR reporting: The case of U.S. banks. *Journal of Business Research* **109**, 362-374, doi:<https://doi.org/10.1016/j.jbusres.2019.12.025> (2020).

20 Grullon, G., Kanatas, G. & Weston, J. *Religion and corporate (mis)behavior* (Houston, TX, 2010).

21 McGuire, S. T., Omer, T. C. & Sharp, N. Y. The impact of religion on financial reporting irregularities. *Accounting Review* **87**, 645-673 (2012).

22 Dyreng, S., Mayew, W. J. & Williams, C. Religious social norms and corporate financial reporting. *Journal of BUsiness Ethics* **111**, 491-518 (2012).

23 Callen, J. L. & Fang, X. Religion and stock price crash risk. *Journal of Financial and Quantitative Analysis* **50**, 169-195 (2015).

24 Adhikari, B. K. & Agrawal, A. Does local religiosity matter for bank risk-taking? *Journal of Corporate Finance* **38**, 272-293, doi:<https://doi.org/10.1016/j.jcorpfin.2016.01.009> (2016).

25 Benjamin, D. J., Choi, J. J. & Fisher, G. Religious Identity and Economic Behavior. *The Review of Economics and Statistics* **98**, 617-637, doi:10.1162/REST_a_00586 (2016).

26 Laurin, K., Shariff, A. F., Henrich, J. & Kay, A. C. Outsourcing punishment to God: beliefs in divine control reduce earthly punishment. *Proceedings of the Royal Society B: Biological Sciences* **279**, 3272-3281, doi:doi:10.1098/rspb.2012.0615 (2012).

27 Bai, J., Lee, E. & Zhang, C. Capital market frictions and human capital investment: Evidence from workplace safety around regulation SHO. *Financial Review* **55**, 339-360, doi:<https://doi.org/10.1111/fire.12227> (2020).

28 Roychowdhury, S. Earnings management through real activities manipulation. *Journal of Accounting and Economics* **42**, 335-370, doi:<https://doi.org/10.1016/j.jacceco.2006.01.002> (2006).

29 MSCI. *MSCI ESG Research: Overview and Products*, <<http://www.msci.com/resources/factsheets/MSCI_ESG_Research.pdf>>

30 Chava, S. & Roberts, M. R. How does financing impact investment? The role of debt covenants. *The journal of finance* **63**, 2085-2121 (2008).

31 Di Giuli, A. & Kostovetsky, L. Are red or blue companies more likely to go green? Politics and corporate social responsibility. *Journal of Financial Economics* **111**, 158-180, doi:<https://doi.org/10.1016/j.jfineco.2013.10.002> (2014).

32 Penick, J. L. *The New Madrid Earthquakes*. (University of Missouri Press, 1981).

33 Smith, B. W., Pargament, K. I., Brant, C. & Oliver, J. M. Noah revisited: Religious coping by church members and the impact of the 1993 midwest flood. *Journal of Community Psychology* **28**, 169-186, doi:<https://doi.org/10.1002/(SICI)1520-6629(200003)28:2><169::AID-JCOP5>3.0.CO;2-I (2000).

34 Zapata, O. Turning to God in Tough Times? Human Versus Material Losses from Climate Disasters in Canada. *Economics of Disasters and Climate Change* **2**, 259-281, doi:10.1007/s41885-018-0029-2 (2018).

1. [↑](#footnote-ref-1)
